# Piriform seizures mediated by the piriform-entorhino-dentate circuit induce brain-wide functional reorganization in mice

**DOI:** 10.1371/journal.pbio.3003577

**Published:** 2026-02-12

**Authors:** Yan Tao, Yuxin Zhao, Wenqi Zhong, Jiajia Zhang, Hongyan Zhu, Xutao Zhu, Zikun Wang, Na Wang, Liqin Yang, Fuqiang Xu, Ruiqi Wu

**Affiliations:** 1 Shanghai Pudong Hospital, Fudan University Pudong Medical Center, State Key Laboratory of Brain Function and Disorders, MOE Frontiers Center for Brain Science, Institutes of Brain Science, Fudan University, Shanghai, China; 2 Department of Radiology, Huashan Hospital, Fudan University, Shanghai, China; 3 Institute of Functional and Molecular Medical Imaging, Fudan University, Shanghai, China; 4 Shenzhen Institutes of Advanced Technology, Chinese Academy of Sciences, Shenzhen, China; Institute for Basic Science, REPUBLIC OF KOREA

## Abstract

Systematic identification of global epileptic reorganization and critical seizure-controlling circuits is essential for comprehending epilepsy pathophysiology and for developing network-guided targeted therapies. The piriform cortex (PC) is a recognized epileptogenic region, but how its hyperactivity reshapes whole-brain dynamics and which specific circuits mediate seizures remains unclear. Through multimodal integration of optogenetics, fMRI, electrophysiology, Ca^2+^ imaging, neural tracing, and circuit-specific manipulation, we mapped the whole-brain dynamics following optogenetic stimulation of PC and identified the fundamental circuit governing piriform seizures. We observed pronounced generalized seizures in mice via repeated optogenetic stimulation of PC *Vglut1*+ neurons. Optogenetic kindling of PC^Vglut1^ induced widespread blood-oxygen-level-dependent (BOLD) signal hyperactivation and resting-state functional connectivity (rsFC) alterations, notably sustained hyperactivation in the lateral entorhinal cortex (Lent) and enhanced PC-Lent rsFC. Chronic elimination of Lent neurons receiving PC projections significantly decreased the Lent-dentate gyrus (DG) rsFC. Disruption of the PC-Lent or Lent-DG circuit effectively suppressed PC-stimulation-triggered seizures and brain-wide hyperactivation. Our findings demonstrate the dominant role of the PC^Vglut1^-Lent^glut^-DG circuit in mediating piriform seizures and driving their resulting brain-wide functional reorganization, offering new insights for targeted epilepsy treatments.

## Abstract 2

Seizures originating from the piriform cortex can lead to widespread alterations in brain function, but the circuits responsible for these effects are not well understood. This study in mice shows that the piriform-entorhino-dentate pathway is a key mediator of brain-wide functional reorganization, providing insights on potential therapeutic targets.

## Introduction

Epilepsy, a prevalent network-level neurological disorder affecting 1% of the global population, manifests as spontaneous recurrent seizures (SRSs) arising from aberrant multi-regional neuronal interactions [1]. Approximately 30% of patients develop medically refractory epilepsy, with temporal lobe epilepsy (TLE) being the most common type [2]. The current therapeutic intractability of TLE imposes substantial socioeconomic burdens, necessitating novel interventions that transcend traditional focus-based approaches [2]. Emerging evidence supports neuromodulation targeting network hubs or key circuits beyond epileptogenic zones as a promising precision strategy [3,4], underscoring the imperative to delineate causal networks and circuits driving epileptogenesis.

The piriform cortex (PC), a center for olfactory processing located at the junction of the temporal and frontal lobes, is a critical epileptic seizure onset zone across species, including humans [5], monkeys [6], and rodents [7,8]. The PC is susceptible to seizure induction through nonspecific electrical stimulation of a pan-neural population and specific optogenetic activation of principal neurons [9–11]. Epileptogenic remodeling in the PC involves small volume, neuronal loss, neural activity impairment, and neurotransmitter dysregulation [10–12]. While previous animal research has predominantly focused on local PC pathology, system-level network dynamics and causal circuit mechanisms are lacking. Anatomically, the PC is interconnected with multiple brain regions, including the entorhinal cortex, amygdala, and orbitofrontal cortex, positioning it within a complex network [13–16]. Human functional imaging studies highlight the PC as a critical hub within the epileptogenic network and the hyperconnectivity of the PC in the epileptic brain [17–19]. Nevertheless, the mechanisms by which the PC’s focal hyperactivity drives brain-wide network dysfunction and the specific circuitry primarily responsible remain unclear. This knowledge gap is partly due to two key challenges: 1) the complexity of dissecting specific circuits within the PC’s intricate connectivity matrix; and 2) the lack of a multimodal integration that bridges cell-type-specific manipulation with cross-scale functional and anatomical readouts.

To address these challenges, we employed a multimodal framework combining optogenetic functional MRI (ofMRI), electrophysiology, in vivo calcium imaging, and viral circuit tracing. ofMRI incorporates high-field MRI and optogenetics, enabling precise control over repeated seizure generation and facilitating a longitudinal in vivo assessment of brain-wide functional activation and reorganization [20,21]. Thus, it allows the identification of epilepsy network hubs and intervention effects. Additionally, the application of gold-standard approaches for direct measurement of epileptiform activity (e.g., electrophysiology), along with the tracing of specific anatomical connections using novel neurotropic viruses, facilitates further identification of critical regions and circuits underlying seizures [22]. Coupled with circuit-specific manipulation, this multimodal framework, using fMRI as an integrative catalyst for cross-scale data [23], enables quantifying causal links between PC focal hyperactivity, network/circuit dysfunction, and behavioral outcomes.

Here, we systematically investigated the circuitry governing the propagation and suppression of piriform seizures through network hub identification and causal validation. By leveraging ofMRI, resting-state fMRI (rsfMRI), and virus tracing, we identified the potential downstream region contributing to the propagation of PC-kindling-induced seizures. We then selectively activated and suppressed this region and its interconnected circuit with the PC to delineate their roles in piriform seizures and blood-oxygen-level-dependent (BOLD) signal changes. Our findings reveal brain-wide functional reorganization in PC-kindled seizures and pinpoint the critical role of the piriform-entorhino-dentate circuit in mediating piriform seizures. This circuit complements the classical Papez circuit in TLE pathophysiology, providing a novel potential therapeutic target for precision treatments.

## Results

### Repeated optogenetic stimulation of *Vglut1*+ neurons in PC induces seizures that spread via synaptic transmission

**Fig 1:**
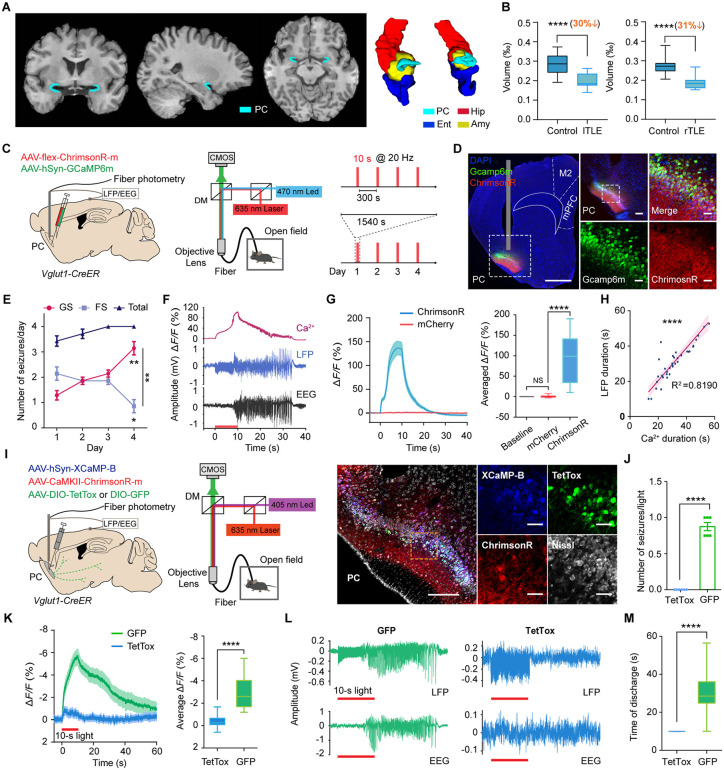
PC atrophy in TLE patients and PC-kindling-induced seizures in mice propagate via synaptic transmission. **(A)** Left, coronal, sagittal, and axial views of PC. Right, a 3D view of the PC and surrounding regions. **(B)** A volumetric comparison of the PC between healthy controls and patients with TLE. A comparison was conducted between the epileptic side ROIs of patients with TLE and the same side of controls (for lTLE: *n* = 12 and 30 in the patient and control groups, respectively; PC-left: unpaired *t*-test, *t*_40_ = 5.490, *P* < 0.0001. For rTLE: *n* = 15 and 30 in the patient and control groups, respectively; PC-right: unpaired *t t*est, *t*_43_ = 7.605, *P* < 0.0001). **(C)** The experimen*t*al scheme of viral injection, synchronous electrophysiological and calcium recordings, and the kindling timeline. **(D)** Left, virus expression in the PC; scale bar: 1 mm. Right, enlarged images from the left dashed square, scale bar: 200 μm; scale bars for enlarged images: 50 μm. **(E)** The number of GSs (Day 4 vs. Day 1, *t*_6_ = 4.596, *P* = 0.0037) and FSs (Day 4 vs. Day 1, *t*_6_ = 2.714, *P* = 0.0349) per day (GS vs. FS on Day 4, *t*_6_ = 4.382, *P* = 0.0047); *n* = 7. **(F)** Representative calcium (top), LFP (middle), and EEG signals (bottom) recorded in the PC during 10-s of light-induced seizures. **(G)** Left, trial-averaged time course of Ca^2+^ signals (∆*F/F*) from the kindled and control groups. Right, an averaged Ca^2+^ signal from the two groups (Kruskal-Wallis test followed by Dunn’s multiple comparison; baseline vs. mCherry, *P* > 0.9999; mCherry vs. ChrimsonR, *P* < 0.0001; n = 32). **(H)** Correlation between Ca^2+^ duration and LFP duration in the PC (Pearson correlation, *R*^2^ = 0.8190, *P* < 0.0001, *n* = 36 from nine mice). **(I)** Viral injections and viral expression in the PC. Scale bar: 200 μm; enlarged images from the dashed square, scale bars: 40 μm. **(J)** Number of seizures (unpaired *t* test, *t*_10_ = 15.65, *P* < 0.0001, *n* = 6). **(K)** Left, *t*rial-averaged time course of Ca^2+^ signals (∆*F*/*F*) from the Te*t*Tox-treated and control groups. Right, averaged Ca^2+^ signals of the two groups (unpaired *t* test with Welch’s correction, *t* = 8.465, *P* < 0.0001, *n* = 24/group). **(L)** Representative LFPs and EEG signals in the TetTox-trea*t*ed and control groups. **(M)** Discharge duration between the two groups (unpaired *t* test with Welch’s correction, *t* = 8.325, *P* < 0.0001, *n* = 24/group). *P < 0.05, **P < 0.01, ****P < 0.0001. Red bars in F, K, and L represent *t*he 10-s light stimulation period. Abbreviations: Amy: amygdala; PC: piriform cortex; EEG: elec*t*roencephalogram; Ent: entorhinal cortex; Hip: hippocampus; LFP: left field potential; lTLE: left temporal lobe epilepsy; rTLE: right temporal lobe epilepsy; TLE: temporal lobe epilepsy; *Vglut1*: vesicular glutamate transporter 1; FS: focal seizure; GS: generalized seizure. The data underlying this Figure can be found in S1 Data.

First, we performed a volumetric analysis of the PC in patients with TLE based on 3D MRI images. The manually segmented, ribbon-shaped PC region, located rostromedial to the amygdala and adjacent to medial temporal regions, including the entorhinal cortex and the hippocampus, was assessed (Fig 1A). We found that the PC volumes in the epileptic hemisphere were significantly decreased in patients with both left- and right-sided TLE (Fig 1B). Specifically, the mean PC volume was reduced by 30% in patients with lTLE (lTLE volume fraction: 0.200‰ ± 0.036‰, control volume fraction: 0.285‰ ± 0.049‰) and by 31% in patients with rTLE (rTLE volume fraction: 0.185‰ ± 0.031‰, control volume fraction: 0.270‰ ± 0.037‰) compared to healthy controls. Moreover, compared to controls, patients showed a significant ~26% volume reduction in the left Hip, while only mild, nonsignificant changes in the Ent, the Amy, and the right Hip (S1 Fig). Severe atrophy in the PC was observed in patients with TLE, suggesting the PC’s important involvement in epilepsy.

Then, we targeted the PC in mice to delineate its role in epileptogenesis. Here, we specifically focused on the anterior PC based on well-established evidence from previous studies by our group and others, which demonstrate that this area has a high propensity for triggering limbic epileptic seizures in both rodents and primates [6,7,24–26]. It is noted that the “PC” refers specifically to the “anterior PC” in the following context.

*Vglut1*+ neurons are dominant among glutamatergic neurons in the cerebral cortex [27]. The number of *Vglut1*+ neurons in the PC was significantly higher than that of *Vglut2*+  neurons (S2A and S2B Fig). Additional analysis of publicly available datasets (Allen Brain, C57BL6J-638850) confirmed strong co-expression of *CaMKIIα* and *Vglut1* in PC neurons, with ~98% of *CaMKIIα*^+^ neurons expressing *Vglut1*, and ~92% of *Vglut1+* neurons expressing *CaMKIIα* (S2C and S2D Fig). We selectively optogenetic kindle *Vglut1*+ neurons (opto-kindling) in *Vglut1-CreER* mice to assess the role of *Vglut1*+ neurons in PC in seizure induction (Fig 1C and 1D). Electroencephalograms (EEGs), local field potentials (LFPs), and Ca^2+^ signals were recorded during the optogenetic experiments. An increasing number of generalized seizures (GSs) was observed during opto-kindling days in PC-kindling mice (Fig 1E). Moreover, kindled mice spent less time in the open arm of the elevated-plus-maze compared to the nonkindled mice (S3 Fig). Obvious epileptiform discharges were accompanied by a significant increase in calcium signals during seizures (Fig 1F and 1G). A significant positive correlation was found between the duration of the calcium and LFP signals (Fig 1H). Additionally, we found that optogenetic stimulation at a mere 1 mW for opto-kindling power (S4A–S4D Fig) and CNO administration at a dose as low as 2 mg/kg for chemogenetic (S4E–S4G Fig) PC^Vglut1^ targeting were adequate to evoke seizures. Moreover, we detected SRSs in more than half of the mice (*n* = 11/19) by re-stimulating PC^Vglut1^-kindled mice after 2 weeks (S1 Movie and S5 Fig). These results demonstrate that opto-kindling of PC *Vglut1*+ neurons readily evoked epileptiform behavior.

Next, we investigated whether the spread of PC-opto-kindling-induced seizures depends on synaptic transmission. AAV-DIO-GFP or DIO-tetanus toxin tight chain (TetTox), which can cleave the synaptic-vesicle-associated VAMP2 protein and block glutamate release [28,29], was injected into the PC (Fig 1I). Following TetTox treatment, seizures were entirely suppressed, and there was a significant reduction in Ca^2+^ response (∆*F/F*) in the mice (Fig 1J and 1K). Epileptiform waves were eliminated after blocking the glutamate release of PC^Vglut1^ neurons (Fig 1L and 1M). These findings suggest that piriform seizure propagation relies on synaptic transmission.

### The Lent exhibited sustained hyperactivation and increased rsFC with the PC in PC-optokindled mice

**Fig 2:**
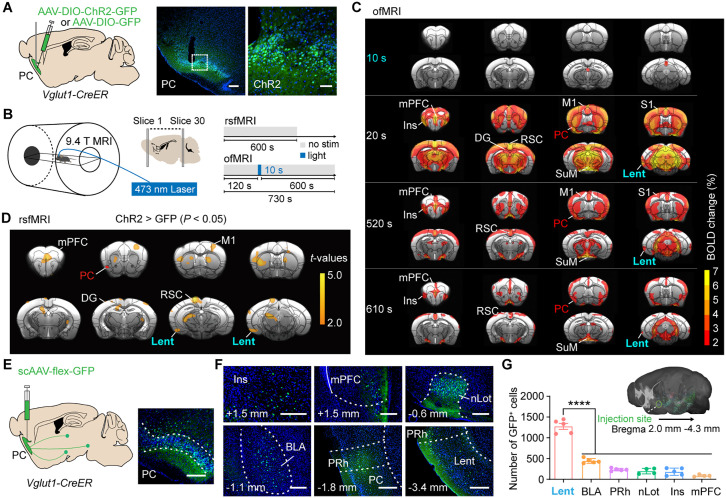
Functional maps in PC-kindled mice reveal that the Lent is functionally activated and reorganized. **(A)** Viral injection and fiber implantation in the PC. Scale bar: 200 μm; scale bar of the enlarged image: 50 μm. **(B)** An experimental scheme of fMRI scanning after PC-kindling. **(C)** BOLD-fMRI activations induced by PC-kindling, *n* = 20/group. **(D)** PC-seed-based functional connectivity alterations between the ChR2-group (*n* = 25) and the GFP-control (*n* = 30). The red-orange blobs indicate significantly increased functional connectivity (ChR2 > GFP, *P* < 0.05). **(E)** Left, scAAV-flex-GFP was injected into the PC. Right, representative GFP expression in the PC. Scale bar: 100 μm. **(F)** Representative labeled neurons in different regions. Scale bars: 100 μm. **(G)** Top, 3D rendering of postsynaptic neurons projecting from the PC. Bottom, quantification of postsynaptic labeled neurons in the ipsilateral hemisphere (one-way ANOVA followed by Dunnett’s multiple comparisons test, the Lent vs. other regions, *P* < 0.0001). Lent: 1,277 ± 72.08, *n* = 5; BLA: 443.0 ± 25.81, *n* = 5; PRh: 234.2 ± 14.30, *n* = 5; nLot: 198.3 ± 33.63, *n* = 4; Ins: 184.2 ± 38.00, *n* = 5; mPFC: 88.50 ± 11.88, *n* = 4. Abbreviations: Ins: Insular cortex; ANOVA: analysis of variance; PC: piriform cortex; BLA: basolateral amygdaloid nucleus; BOLD-fMRI: blood-oxygen-level-dependent functional magnetic resonance imaging; ChR2: channelrhodopsin-2; fMRI: functional magnetic resonance imaging; GFP: green fluorescent protein; Lent: lateral entorhinal cortex; M1: primary motor cortex; mPFC: medial prefrontal cortex; nLot: nucleus of the lateral olfactory tract; PAG: periaqueductal gray; PRh: perirhinal cortex; RSC: retrosplenial cortex; S1: primary somatosensory cortex; SuM: supramammillary nucleus. The data underlying this Figure can be found in S1 Data.

Subsequently, we investigated the circuit mechanisms underlying piriform seizures. Whole-brain functional and structural analyses provide a framework for characterizing circuit organization. To examine how PC-kindling affects brain-wide activation and functional connectivity, we injected Cre-dependent channelrhodopsin-2 (ChR2) into the PC and collected the BOLD signal with a 9.4 T MRI scanner (Fig 2A and 2B). We measured the dynamic activity triggered by 10-s opto-stimulation using ofMRI after stable seizures (Fig 2C). Robust, brain-wide BOLD responses were observed in the bilateral hemispheres following the opto-stimulation of PC^Vglut1^ neurons. This phenomenon was consistent with the occurrence of seizures several seconds after opto-stimulation (S2 Movie). Several regions, including the Lent, medial prefrontal cortex (mPFC), insula, sensorimotor cortex, PC, and supramammillary nucleus (SuM), exhibited sustained BOLD hyperactivation for 10 min. Furthermore, c-Fos-iDISCO+ was applied to confirm BOLD activation during epileptic seizures (S6A–S6C Fig). An overlap was observed between the ofMRI and c-Fos iDISCO+ maps (S6D Fig). The Dice coefficient was used to quantify the similarity between two independent activation maps [30]. The Dice coefficient was 0.61 (*P* < 0.005), revealing a significant similarity between the fMRI and c-Fos maps.

To capture the intrinsic functional reorganization after PC-kindling, we applied seed-based rsfMRI to measure resting-state functional connectivity (rsFC) alterations. The connection map illustrated significant rsFC changes (*P* < 0.05) after PC-kindling (Fig 2D). The rsFC between the PC and several distant regions, including the Lent, mPFC, dentate gyrus (DG), and sensorimotor cortex, was significantly enhanced. These elevated rsFC regions mostly corresponded to long-lasting activated regions.

Additionally, to determine the direct downstream projections of PC *Vglut1*+ neurons, the scAAV2/1-flex-GFP virus, an anterograde monosynaptic tracing virus [31,32], was injected into the PC of *Vglut1-CreER* mice (Fig 2E). Neurons in several remote regions, including the Lent, basolateral amygdaloid nucleus (BLA), perirhinal cortex (PRh), insular cortex, the nucleus of the lateral olfactory tract (nLot), and mPFC, were labeled (only the top six regions within Bregma +2.0 mm to −4.3 mm are illustrated in Fig 2F and 2G, normalized data are illustrated in S7 Fig). The highest number of labeled neurons in the Lent suggests that the Lent has a dominant structural connection to the PC.

Collectively, our functional and structural mapping indicates that the Lent, the primary direct projection region of the PC, exhibits sustained robust activation following 10 s of PC-kindling as well as increased rsFC with the PC in PC-kindled mice. These data suggest that the PC-Lent circuit may play an important role in the propagation of PC-triggered seizures.

### The PC-Lent circuit was necessary for the generation of PC-induced epileptic seizures

**Fig 3:**
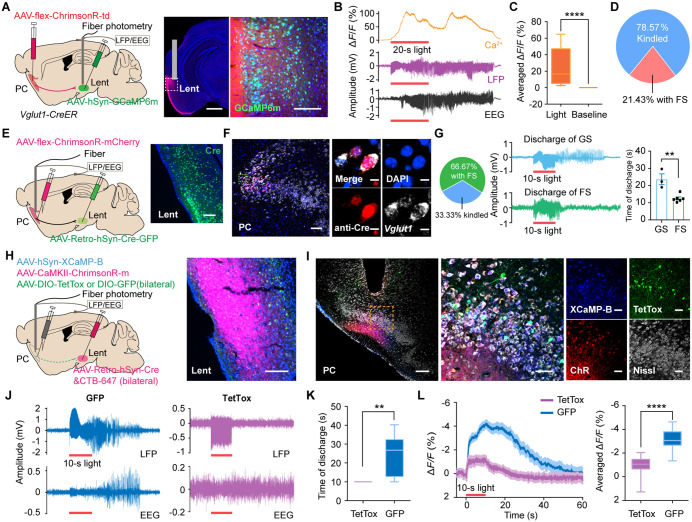
The PC-Lent pathway mediates the generation of PC-kindling-induced seizures. **(A)** Left, a schematic diagram of viral injections; scale bar: 1 mm. GCaMP6m expression in the Lent; scale bar: 200 μm. **(B)** Representative calcium, LFP, and EEG signals during Lent stimulation. Red bar: 20-s light stimulation. **(C)** Ca^2+^ signal changes in the Lent (Mann–Whitney test, *P* < 0.0001, *n* = 13). (D) 78.57% of the 14 mice were kindled. **(E)** A schematic diagram of viral injections in C57 mice; scale bar: 100 μm. **(F)** Left, the expression of Cre and *Vglut1* in the ipsilateral PC; scale bar: 100 μm; and enlarged image, scale bars: 10 μm. **(G)** Left, 33.33% of the nine mice were kindled. Middle, representative discharge signals of GS (top) and FS (bottom) evoked by opto-stimulation. Right, comparison of discharge duration. Red bar: 10-s light stimulation. **(H)** Left, viral and CTB injections in C57 mice. Right, CTB expression in the Lent; scale bar: 100 μm. **(I)** Left, XCaMP-B, ChrimsonR, and TetTox expression in the PC; scale bar: 200 μm. Middle, enlarged image; scale bar: 200 μm. Right, images of split channels; scale bars: 50 μm. **(J)** Representative LFP (top) and EEG (bottom) signals in control (left) and TetTox-treated (right) mice. Red bar: 10-s light stimulation. **(K)** The duration of discharge (unpaired *t t*est, *t*_10_ = 3.218, *P* = 0.0092, *n* = 6). **(L)** Averaged calcium signal (∆*F*/*F*) in con*t*rol and TetTox-treated mice (Mann–Whitney test, *P* < 0.0001, *n* = 24). **P < 0.01, ****P < 0.0001. Red bar: 10-s light stimulation. Abbreviations: PC: piriform cortex; Lent: lateral entorhinal cortex; *Vglut1*: vesicular glutamate transporter 1. The data underlying this Figure can be found in S1 Data.

The above results motivated us to investigate the role of the PC-Lent circuit in piriform seizures. We stimulated dense fibers from the PC in the Lent, after injecting AAV-flex-ChrimsonR in the PC (Fig 3A). Stimulation of PC terminals in the Lent evoked GSs in 78.57% of the mice with increased calcium signals (Fig 3B–3D). To identify which PC neurons projecting to the Lent were responsible for seizure generation, we injected retroAAV-Cre into the unilateral Lent and flex-ChrimsonR into the ipsilateral PC in wild-type mice (Fig 3E). Cre^+^ neurons in the bilateral PC were overlaid with ChrimsonR (S8A and S8B Fig). Moreover, 95% of the labeled Cre^+^ neurons are *Vglut1*^+^ neurons (Fig 3F). Opto-kindling of PC neurons that project to the Lent evoked GSs in three of the nine animals (Fig 3G). In the other six animals, optogenetic stimulation elicited only focal after-discharge in the PC without evolving into generalized seizures (Fig 3G). The three kindled mice had a significantly longer discharge duration than the other six mice with only focal seizures (Fig 3G). Given the evidence of PC projections to bilateral Lent and corresponding bilateral activation in both c-Fos and fMRI maps (Figs 2C, S8C and S9), we injected retroAAV-Cre into the Lent and DIO-TetTox into the PC bilaterally to obstruct the connection between the PC and Lent. AAV-CaMKIIα-ChrimsonR and hSyn-XCaMP-B viruses were then injected into the unilateral PC (Fig 3H and 3I). Inhibition of the PC-Lent pathway abolished the generation of seizures and blocked epileptiform discharges (Fig 3J and 3K), as well as weaker calcium signals (Fig 3L).

Moreover, we examined other regions’ role in PC-kindling-induced seizures, including the two strong downstream regions (the Prh and the BLA) and the two regions with strong BOLD activation but lack direct anatomical projections to the PC (the M1 and the RSC). We found that inhibiting either the bilateral PC-BLA or PC-Prh pathways with TetTox did not prevent the occurrence of seizures after the 4 days of PC-opto-kindling, although it partially suppressed them (S10A and S10B Fig). Additionally, inhibiting either bilateral M1 or bilateral RSC resulted in minimal or no seizure suppression (S10C and S10D Fig). Statistical data showed that the number of seizures was significantly greater than the number observed after the PC-Lent inhibition (S10E and S10F Fig). These results indicated that the PC-Lent pathway plays a predominant role in PC-triggered seizure propagation.

**Fig 4:**
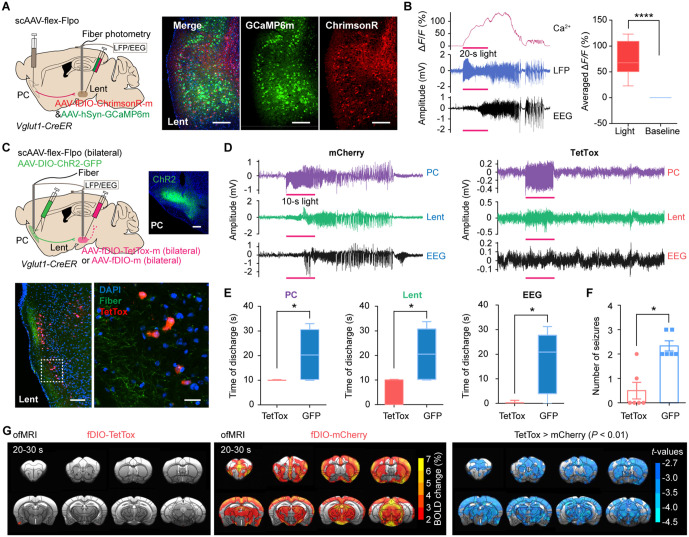
Lent neurons that receive PC projections control PC-kindling-induced seizures and BOLD hyperactivation. **(A)** A schematic diagram of viral injections; scale bars: 200 μm. **(B)** Left, representative calcium (top), LFP (middle), and EEG (bottom) signals from one mouse in the Lent. Red bar: 20-s light stimulation. Right, statistical analysis of calcium signal changes (Mann-Whitney test, *P* < 0.0001, *n* = 6) **(C)** Top, a schematic diagram of viral injections; scale bar: 200 μm. Bottom, scale bars: 100 μm and 20 μm (the enlarged image). **(D)** Representative LFP and EEG traces recorded from a mCherry-treated mouse (left) and a TetTox-treated mouse (right). Red bar: 10-s light stimulation. **(E)** Duration of the LFP discharge and EEG (Mann–Whitney test, *P* = 0.0152 in the PC; *P* = 0.0108 in the Lent; *P* = 0.0152 in the EEG; *n* = 6). **(F)**, Number of seizures (Mann–Whitney test, *P* = 0.0108, *n* = 6). **(G)** BOLD activations induced by PC-kindling in the fDIO-TetTox (*n* = 16; left) and fDIO-mCherry (*n* = 25; middle) groups. Right, BOLD signal alterations were compared between the two groups. Blue blobs overlaid on reference structural images indicate significant alterations (fDIO-TetTox > fDIO-mCherry, *P* < 0.01). *P < 0.05, ****P < 0.0001. Abbreviations: PC: piriform cortex; BOLD: blood-oxygen-level-dependent; EEG: electroencephalogram; Lent: lateral entorhinal cortex. The data underlying this Figure can be found in S1 Data.

To overcome limitations of fiber stimulation (such as the nonselective activation of traversing fibers), we injected a Cre-dependent Flpo virus into the PC and Flpo-dependent fDIO-ChrimsonR into the Lent to exclusively activate PC-targeted Lent neurons (Fig 4A). All six mice exhibited seizure-like behaviors (discharge duration: 49.85 ± 3.148 s) and significantly increased changes in calcium signals (73.90% ± 6.305%) (Fig 4B). Moreover, 87.84% of fDIO-labeled neurons were glutaminergic neurons, while 3.56% were GABAergic neurons (S11 Fig). These results indicate that *Vglut1*+ neurons in the PC project predominantly to glutaminergic neurons in the Lent, with readily evoked GSs.

Furthermore, we bilaterally injected scAAV-flex-Flpo into the PC and fDIO-TetTox into the Lent to obstruct the downstream circuits of the PC-Lent (Fig 4C). In addition to significantly shortening the duration of EEG and LFP discharges compared to the control group, TetTox significantly reduced epileptic activity in the PC (Fig 4D and 4E). Inhibition of the PC-Lent circuit significantly diminished seizure incidence (Fig 4F). Moreover, the hyperactivation of BOLD signals induced by PC^Vglut1^-kindling was eliminated after the inhibition of PC-targeted Lent neurons (Fig 4G). These results demonstrate that targeted Lent blocking limits PC^Vglut1^-kindling-induced seizures, highlighting the critical role of the PC^Vglut1^-Lent^glut^ circuit in generating PC-triggered epileptic seizures.

### The Lent-DG circuit mediated the propagation of PC-kindling-induced seizures

**Fig 5:**
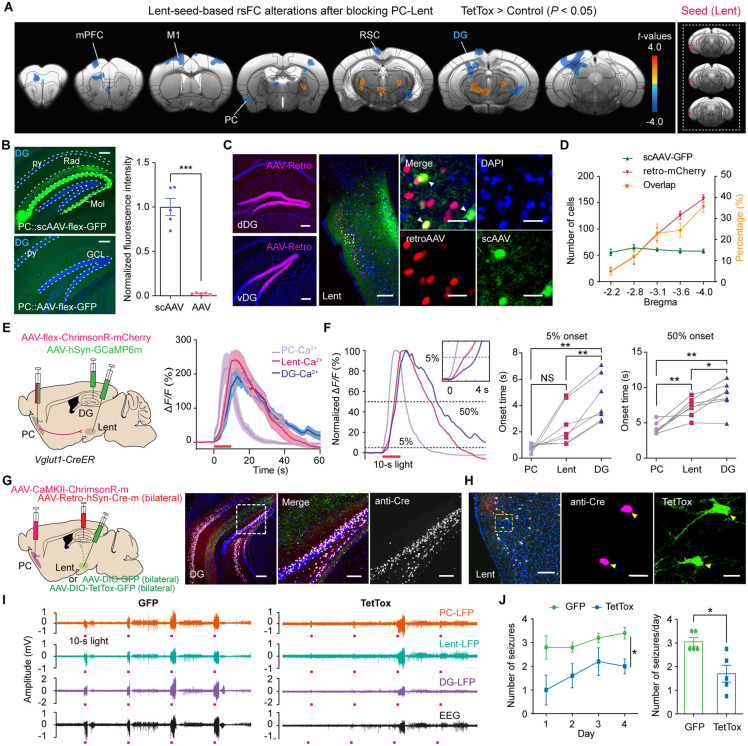
The Lent-DG pathway contributes to PC-kindling-induced seizures. **(A)** Lent-seed-based rsFC alterations after blocking the synaptic transmission of PC-targeted Lent neurons. **(B)** Left, signals in DG fibers after injecting scAAV-flex-GFP (Top) and AAV-flex-GFP (Bottom) into the PC; scale bars: 200 μm. Right, normalized fluorescence intensity in the DG (unpaired *t* test with Welch’s correction, *t* = 10.06, *P* = 0.0005, *n* = 5). **(C)** Left, viral injections in the DG. Right, GFP^+^ and mCherry^+^ cells in the Lent; scale bar: 200 μm; an enlarged image, scale bars: 25 μm. **(D)** Number of GFP^+^ and mCherry^+^ cells and percentage of co-labeled cells. *n* = 5. **(E)** Left, viral injections in the ipsilateral PC, Lent, and DG. Right, Ca^2+^ signals from the PC, Lent, and DG. *n* = 7. **(F)** Times to reach the 5% (repeated measures one-way ANOVA followed by Tukey’s post hoc test, PC vs. Lent, *P* = 0.0955; PC vs. DG, *P* = 0.0061; Lent vs. DG, *P* = 0.0018) and 50% (PC vs. Lent, *P* = 0.0054; PC vs. DG, *P* = 0.0016; Lent vs. DG, *P* = 0.0340) peaks of Ca^2+^ response. *n* = 7. **(G)** Left, the experimental scheme of viral injections. Right, viral expression in the DG; scale bar: 200 μm. Enlarged image of the DG; scale bars: 100 μm. **(H)** Cre^+^ and TetTox^+^ cells expression in the Lent; scale bar: 100 μm. Yellow arrows indicate co-labeled cells; scale bars: 20 μm. **(I)** Representative LFP and EEG signals in the TetTox-treated (left) and control (right) groups. **(J)** Left, the number of seizures in the two groups (repeated measures two-way ANOVA followed by Bonferroni’s post hoc test, *F*_1_ = 10.881, *P* = 0.0109, *n* = 5). Right, the average number of seizures per day (Mann–Whitney test, *P* = 0.0317, *n* = 5). *P < 0.05, **P < 0.01, ***P < 0.001. Red bars in E, F, and I represent the 10-s light stimulation. Abbreviations: PC: piriform cortex; DG: dentate gyrus; Lent: lateral entorhinal cortex. The data underlying this Figure can be found in S1 Data.

To identify how PC seizures transmitted by the PC-Lent pathway propagated further, we next investigated the downstream circuit. By analyzing Lent-seed-based rsFC, we observed a significant decrease in functional connectivity between the DG and Lent after blocking neurotransmitter release from PC-targeted Lent neurons (Fig 5A). In addition, we demonstrated increased rsFC between the PC and DG in PC-kindled mice (Fig 2D). These findings suggest that the DG is functionally involved in piriform seizures. By injecting scAAV-GFP into the PC, we noticed dense fiber distribution over the molecular layer of the DG, suggesting a strong transsynaptic projection between the PC and DG (Fig 5B). We injected the scAAV-flex-GFP virus into the PC and the retroAAV-hSyn-mCherry virus into the dorsal and ventral DG. The PC-projecting and DG-targeting neurons converged on the superficial layer of the Lent (Fig 5C and 5D). CTB tracing results further confirmed the PC-Lent-DG circuit (S12 Fig). Then, we simultaneously recorded Ca^2+^ signals in the PC, Lent, and DG to capture the ictal propagation profile in these regions during seizures, and found that ictal propagation follows the PC-Lent-DG sequence, with each step delayed by 2–4 s (Fig 5E and 5F). We also found that the duration of the calcium responses was increased from day 1 to day 4 across the PC, the Lent, and the DG (S13 Fig). This finding suggests that the shift toward generalized seizures involves a progressive strengthening of activity across all nodes throughout the PC-Lent-DG circuit.

The above results indicate that the Lent-DG connection may be involved in the propagation and control of PC seizures transmitted by the PC-Lent pathway. Furthermore, we bilaterally injected retroAAV-Cre into the DG and DIO-TetTox into the Lent to inhibit the Lent-DG circuit and to determine whether disruption to the Lent-DG circuit contributed to seizure relief (Fig 5G and 5H). We noticed that TetTox-labeled neurons projected dense fibers to the DG. Moreover, the number of seizures decreased significantly in the blocked group (Fig 5I and 5J). These results revealed that Lent-DG inhibition is beneficial for the relief of PC-triggered seizures.

Collectively, by using a multimodal framework, this study identified the PC-Lent-DG circuit as essential for the propagation and control of piriform seizures.

## Discussion

Here, we found that optogenetic stimulation of PC *Vglut1*+ neurons in mice readily induces seizures, with their propagation dependent on synaptic transmission (Fig 1). We observed sustained BOLD hyperactivation in the Lent—the primary direct projection region of the PC—for about 10 min following PC-kindling and noted enhanced rsFC between the PC and Lent in PC-optokindled mice (Fig 2). By manipulating the PC^Vglut1^-Lent^glut^ circuit in a targeted manner, we pinpointed its essential role in mediating piriform seizures and brain-wide BOLD hyperactivations (Figs 3 and 4). Furthermore, blocking PC-Lent transmission significantly decreased rsFC between the DG and Lent, and chronic elimination of the Lent-DG synaptic transmission effectively alleviated PC-induced seizures (Fig 5). These findings indicate a brain-wide functional reorganization following piriform seizures, emphasizing the critical role of the PC-Lent-DG circuit in their mediation.

### The PC is a critical epileptogenic zone

Optogenetic kindling of PC *Vglut1*+ neurons in mice readily induced seizures, consistent with the established epileptogenic nature of the deep PC area (known as ‘area tempestas’), which is highly prone to triggering limbic epileptic seizures in rodents and primates [24,33]. Compared to traditional electrical kindling, optogenetic kindling offers advantages, including spatiotemporal control of selective cells and artifact-free electrophysiological recording [10,20,34,35]. The challenges associated with SRS generation are the main criticism of the kindling model [20,36]. In this study, over half of the re-optokindled mice exhibited SRSs and continuous interictal epileptic discharges. Even low-intensity optogenetic stimulation (1 mW) or a low dose of chemogenetic CNO (2 mg/kg) applied to the PC sufficed to induce GSs, reinforcing the vulnerability of the PC to seizures via targeted manipulation. We also revealed that anxiety-like behavior was elevated following PC-opto-kindling, a phenomenon comorbid with epilepsy [37]. We found significant PC atrophy in patients with TLE, indicating its important involvement in TLE. This volume reduction may be a consequence of seizure-induced excitotoxicity. The result that atrophy in the Ent, the Amy, and the Hip was less severe than the pronounced ∼30% volume loss in the PC in our cohort of TLE patients underscores the particular vulnerability of the PC to epileptic pathology. Besides, recent studies underscore the PC’s crucial role in TLE regulation across rodents and humans. Selective silencing of PC pyramidal neurons diminished seizure generation in a TLE mouse model [38]. Retrospective clinical studies indicate that more extensive PC resection offers greater TLE relief [39–41].

### Brain-wide reorganization and the PC-Lent-DG circuit’s governing role in piriform seizures

Seizure propagation is dependent on the underlying anatomical connections [42–44]. The propagation of focal cortical seizures relies on axonal pathways, with activity propagating to specific regions via synaptic transmission [20]. Advances in neuroimaging have provided valuable insights into seizure propagation and functional reorganization [20,45]. Our ofMRI results revealed a rapid, brain-wide, robust BOLD response following PC^Vglut1^-kindling, indicating high efficiency in information communication within the piriform seizure network. In addition, our rsfMRI data revealed increased intrinsic functional connectivity between the PC and other limbic regions, such as the Lent and DG, suggesting that functional reconfiguration occurs in the context of piriform seizures. Alterations in synaptic plasticity or an “excitation-inhibition” imbalance may contribute to these connectivity changes. The enhanced rsFC between the PC and mPFC in PC-kindled mice suggests a potential link between the PC and not only TLE but also frontal epilepsy. Notably, although a light anesthesia regimen combining isoflurane and dexmedetomidine (two agents with opposite effects on vasodilation and vasoconstriction) was employed in our study, the experimental conditions under light anesthesia may still partially influence brain activation and network dynamics [46,47]. Additionally, fMRI is limited in its ability to capture fast neuronal events, constrained by the inherently slow nature of the hemodynamic response and the 2-s temporal resolution of our acquisition. Nevertheless, integrating fMRI with novel neural tracing techniques helped identify the downstream nodes of the Lent and DG, revealing them as critical hubs within a broader network in the PC-kindling-induced seizure propagation.

Remarkably, a brief optogenetic stimulation of the PC-Lent circuit triggered GSs. The reciprocal connectivity between the PC and Lent may exacerbate seizures [48]. During seizures, the hyperactivation of the Lent might reinforce and sustain the initial epileptic activity in the PC. This reciprocal connection could explain why blocking glutamate release from PC’s terminals in the Lent with TetTox not only prevents forward propagation but also leads to the observed reduction in PC’s activity. We mapped the PC^Vglut1^-Lent^glut^-DG circuit, which exhibited robust anatomical connectivity through Lent layers II–III. Note that the primary target of the PC projections to the Lent was proximal to the PRh. The DG can process information transmitted from the entorhinal cortex through the medial and lateral perforant pathways [49]. DG mossy axons projected from the granule cells to the CA3, with subsequent connections to pyramidal cells in the CA1 through Schaffer collaterals [50]. This trisynaptic, excitatory loop conveyed information back to the entorhinal cortex [51]. Lent and DG have been implicated in seizure generation, propagation, and regulation [52–54]. Recurrent excitation within the PC-Lent-DG and hippocampus-Lent-PC circuits likely amplifies seizures. The piriform-entorhinal-hippocampal trisynaptic loop has primarily been reported concerning spatial memory [55]. However, no existing literature explicitly delineates the role of the PC-Lent-DG circuit in regulating PC-triggered seizures. Here, we demonstrate that the PC-Lent-DG circuit mediates piriform seizures through the integration of specific manipulations, causal validation, advanced cross-scale neuroimaging, and seizure behavioral assessments (Racine scale).

### Targeting the PC-Lent-DG circuit holds promise for seizure relief

The concept of precision medicine and therapy for epilepsy has gained attention in recent years [56,57]. Neuromodulation, which targets key network hubs relevant to the propagation of epileptic activity outside the epileptogenic focus, has been demonstrated to effectively control seizures in patients [3,58]. Targeted circuit interventions for epilepsy are preferred over global approaches to avoid potential side effects [3,59]. Our findings using multichannel Ca^2+^ imaging delineated a sequential epileptic transmission pathway from the PC to the Lent and further to the DG, with each synaptic transfer exhibiting several-second latencies. It is insufficient to capture fast, monosynaptic events, given the slow kinetics of the GCaMP indicator (half-rise time of ~10 ms) [60] and the 30 Hz sampling rate of our fiber photometry. These timed inter-regional delays establish a therapeutically exploitable temporal window for targeted modulation and provide an opportunity to modulate piriform epilepsy with a temporal resolution of seconds. Crucially, we observed that a disruption of the synaptic transmission from the PC to the Lent or from the Lent to the DG effectively blocks or alleviates PC-triggered seizures and brain-wide hyperactivation. The generalized seizures appear to depend on the recruitment and hyperactivity of this entire circuit, which intensifies the signal from the PC and propagates into more extensive downstream networks subsequently. Neuromodulation that targets specific circuits with appropriate response times holds increased potential for effective seizure suppression. Given that different electrical stimulation frequencies may selectively engage distinct neuronal subtypes [61–65], cell-type-specific circuit studies hold promise to optimize electrical stimulation paradigms for clinical epilepsy neuromodulation. There is promise in leveraging closed-loop stimulations within the specific PC-Lent-DG circuit for the treatment of piriform epilepsy.

The present study characterized the role of the PC^Vglut1^-Lent^glut^-DG circuit in mediating piriform seizures. Unlike classical hippocampal output pathways, such as the Papez circuit that spreads seizures from the hippocampus in TLE, our newly identified PC-Lent-DG input pathway transmits epileptic activity into the hippocampus. We propose that the inclusion of the piriform axis (PC-Lent-DG), alongside the Papez circuit, should be considered in epilepsy neuromodulation or treatments. However, future studies investigating the PC-Lent-DG circuit using chronic epilepsy models (e.g., kainic acid or pilocarpine) are essential for fully elucidating its role in epilepsy. A limitation of this study is that only the anterior PC was investigated. Given that the anterior and posterior subdivisions of the PC exhibit distinct functions and projection patterns [66–68], future investigation should be performed to explore their differential roles in regulating epileptic seizures. Additionally, it is essential to recognize that seizure propagation involves both synaptic and nonsynaptic mechanisms that evolve with seizure progression [44,69].

## Materials and methods

### Ethics statement

Human MRI data collection for this study was approved by the Huashan Hospital Institutional Review Board (no. 2021-917). All participants or their guardians gave written informed consent to use their anonymized MRI images and clinical data for research purposes. The study complied with the guidelines for conducting research involving human subjects as established at Huashan Hospital. All animal experiments were approved by the Fudan University Animal Care Committee and were conducted following the guidelines and regulations designed by the National Institutes of Health Guide for Care and Use of Laboratory Animals (no. DSF-2020-056). The study adhered to the principles of the Declaration of Helsinki.

### Human subjects

We retrospectively collected pre-surgical, high-resolution, 3D, T1-weighted MRI images of 27 (13 female) patients with TLE who underwent temporal lobe surgeries between June 2020 and September 2023 in the Huashan Hospital. T1 MRI images were acquired on a 3T Philips Ingenia scanner at the Department of Neurosurgery in Huashan Hospital with the following parameters: echo time (TE) = 2.9 ms; repetition time (TR) = 6.5 ms; field of view (FOV) = 240 × 240 × 160 mm^3^; and matrix = 288 × 288 × 160. TLE was diagnosed by a multidisciplinary epilepsy management team before surgery based on the guidelines of the lLAE Commission on Classification and Terminology. Patients were split into left-sided and right-sided TLE groups (namely, lTLE and rTLE), according to their epileptic focus and surgery hemisphere. Only patients aged 18–40 years old were included in this study, to avoid the potential influence of age-related confounding factors. The mean age of the patient group was 29.1 year (standard deviation; SD = 6.7). In this study, 30 (16 female) healthy controls without any prior history of a neurological or psychiatric illness were prospectively recruited at the Huashan Hospital. Their mean age was 30.0 year (SD = 5.4). No significant age (*P* = 0.60) or gender (*P* = 0.70) differences were detected between the TLE and control groups.

### Animals

The *Vglut1-CreER* line mice (Biocytogen, China) were gifted from Dr. Fuqiang Xu. Young male adult C57BL/6 mice were purchased from GemPharmatech. All mice were housed under a 12-hour light/dark cycle, with ambient temperature controlled at 22 ± 2 °C and humidity maintained at 50% ± 10%. They had unlimited access to food and water. Every effort was made to minimize the number of animals used and their suffering.

### Viral injections and optic fiber/electrode implantation

Mice were anesthetized using isoflurane (1.0%−1.5%) and secured in a stereotaxic frame (RWD, Shen Zhen, China). We performed viral injections and optic fiber/electrode implantation as described in our previous studies [24,70]. Notably, 100 μm optical fibers were used in fMRI experiments to reduce MRI artifacts. Post-surgery, iodine and lidocaine gel were applied to the surgical site immediately and again 24 hours later to alleviate pain. Additionally, ceftriaxone sodium (180 mg/kg) was administered intraperitoneally for three consecutive days.

All coordinates for the virus microinjections, measured from the bregma, following Paxinos and Franklin’s Mouse Brain Atlas (second edition, 2001), are described below: AP +1.54 mm, ML −2.54 mm, DV −4.74 mm for the PC; AP −2.00 mm, ML −1.25 mm, DV −2.00 mm for the dorsal DG; AP −2.80 mm, ML −1.90 mm, DV −2.25 mm for the ventral DG; AP −1.82 mm, ML −4.10 mm, DV −4.00 mm for the Prh; AP −1.50 mm, ML −3.30 mm, DV −4.72 mm for the BLA; AP 0.01 mm, ML −1.00 mm, DV −1.25 mm for the M1; AP −1.06 mm, ML 0.337 mm, DV 1.00 mm for the RSC; and AP −3.08 mm, ML −4.44 mm, DV −4.40 mm for the Lent. The coordinates provided here target the anterior portion of the PC.

### Viral vectors and drug

AAV-DIO-hM3Dq-mCherry, AAV-EF1α-DIO-ChR2-GFP, AAV-EF1α-DIO-GFP, AAV-flex-ChrimsonR-mCherry, AAV-CaMKIIα-ChrimsonR-mCherry, AAV-flex-ChrimsonR-tdTomato, AAV-fDIO-ChrimsonR-mCherry, AAV-fDIO-TetTox- mCherry, AAV-DIO-TetTox-GFP, AAV-hSyn-TetTox- mCherry, AAV-Retro-hSyn-Cre-mCherry, AAV-Retro-hSyn-Cre, scAAV-flex-GFP, scAAV-flex-Flpo, and AAV-hSyn-GCaMP6m were purchased from Taitool Bioscience (Shanghai, China). AAV-Retro-hSyn-Cre-GFP was obtained from BrainVTA (Wuhan, China). AAV-XCaMP-B and AAV-Retro-hSyn-mCherry were purchased from Braincase (Shenzhen, China). The viral titer of scAAV and AAV-Retro was 2–4 × 10^13^ v.g./mL, while the other viral titers were 2–4 × 10^12^ v.g./mL. Furthermore, 110–200 nL of viral vector was injected in each site. Virus-injected mice were allowed to recover and express the virus for at least 4 weeks post-surgery.

CTB-555 (Thermo Invitrogen, C34776) and CTB-647 (Thermo Invitrogen, C34778) were dissolved to a concentration of 2%. CNO (Tocris, #4936) was dissolved in sterile saline to prepare a final dose of 2 mg/kg. Tamoxifen (Sigma-Aldrich, T5648) was dissolved in sterile corn oil (Macklin, C805618) to prepare a working stock solution of 20 mg/mL. Mice were injected subcutaneously with tamoxifen (0.15 mL per injection) once a day for 5 days.

### Optogenetic experiments

A 473 nm laser was used to activate ChR2-labeled neurons (20 Hz, 5 ms per pulse, duration 10-s, four times per day, 5 min apart), whereas a 635 nm laser (Changchun New Industries) was used to activate ChrimsonR-labeled neurons (20 Hz, 5 ms per pulse; 10 ms per pulse for fiber stimulation). The intensity of the light delivered to the brain was 3 mW. Due to the strong diffusion of red light compared to blue light, ChR2 was utilized in the fMRI experiments. All mice used in fMRI and specific manipulation experiments received a full 4-day kindling procedure.

### Optogenetic fMRI and resting-state fMRI

Once mice exhibited ≥ 2 generalized seizures during the 4 kindling sessions, they were considered “kindled”, and thus included in the subsequent fMRI experiments. Ten kindled mice with blue light stimulation were included in imaging studies. The procedures for animal anesthesia and body temperature maintenance have been described previously [24,71,72]. In brief, anesthesia was induced with a bolus of dexmedetomidine (0.02 mg/kg, i.p.) and maintained with continuous infusion of dexmedetomidine (0.1 mg/kg/h, i.h.) combined with 0.5%−0.8% isoflurane. MRI data were obtained by a uMR 9.4T scanner (United Imaging 94/30, Shanghai, China). An 86-mm diameter volume coil was used for transmission, and a customized single-loop surface coil (14 × 18 mm^2^) was utilized as an RF receiver. Anatomical images were acquired using a fast spin echo sequence with the following parameters: TE = 39 ms; TR = 3,000 ms; matrix size = 256 × 256; FOV = 19 × 15 mm^2^; and slice thickness = 0.35 mm. BOLD-fMRI data were acquired using an echo planar imaging (EPI) sequence with the following parameters: TE = 16 ms, TR = 2,000 ms, matrix size = 96 × 96, FOV = 19 × 15 mm^2^, slice number = 30, and slice thickness = 0.35 mm; there were 310 repetitions for rsfMRI and 365 repetitions for ofMRI. During ofMRI, 10-s blue light (20 Hz, 5 ms, and 3 mW) was delivered to the PC via an optical fiber at 61–65 frames.

### Seizure behavioral analysis

Mice were gently placed into the center of the plastic box (42 × 42 × 30 cm) and allowed to freely move until the end of the test. Seizure behaviors were scored by an investigator who was blinded to the experimental design. Following the Racine scale, we classified seizure severity into stages 1–6 with minor modifications [73]: stage 1, staring, mild facial twitching; stage 2, head nodding, back or turn circles; stage 3, single forelimb clonus; stage 4, clonus in both forelimbs, tail stiffing; stage 5, rearing and falling on the side; and stage 6, running, jumping seizures. Stages 1–3 were considered FSs, whereas stages 4–6 were considered GSs.

### Elevated-plus-maze test

The elevated-plus-maze (EPM) test was performed 30 min after kindling. The EPM consists of two open arms (25 × 5 cm), two enclosed arms (25 × 5 cm), and a central platform (5 × 5 cm). Each mouse was allowed to explore freely for 5 min in the EPM. After each trial, the devices were cleaned with 75% ethyl alcohol. All animal behaviors were recorded and analyzed with ANY-maze (Stoelting, USA).

### Fiber photometric recording and data analysis

Calcium signals were recorded throughout the behavioral test using a tri-color, multichannel, fiber photometric system (ThinkerTech, Nanjing, China) at a sampling rate of 30 Hz as previously described [24]. The GCaMP6m and XCaMP-B signals were recorded using 470 and 405 nm LED channels, respectively [74]. The optogenetic 635 nm laser was connected to a 580 nm LED channel for local stimulation. To preserve the authenticity of the signals recorded during seizures, isosbestic control correction was not performed [75]. Changes in fluorescence (∆*F*/*F*) were calculated as (*F*–*F*_0_)/ *F*_0_, where *F*_0_ is the average baseline fluorescence.

### LFP and EEG recordings

The multichannel signals were digitized at a rate of 1 kHz with 0.1 Hz high-pass and 300 Hz low-pass filtration preceding acquisitions (OmniPlex, Plexon, USA). Four-channel tethered EEG devices (8401-HR, Pinnacle, USA) and video recordings were simultaneously performed in the free-moving mice. The EEG recordings were amplified × 100 using amplifiers (8400-K3, Pinnacle, USA) with 1,000 Hz low-pass filtration preceding acquisition (Sirenia Acquisition, USA) to detect seizure events (Sirenia Seizure Pro, Pinnacle, USA). All mice were maintained under a 12-hour light/dark cycle with food and water ad libitum. The Fourier transform was utilized to extract frequency domain information from the LFP and EEG signals.

Discharges with duration >15 s, amplitude >3 times the baseline, and frequency >1 Hz were defined as a seizure event. Optogenetically evoked epileptiform discharges were identified as seizure-like events that occurred in direct response to the optical stimulation, typically within seconds of its onset. Spontaneous epileptiform discharges were defined as events that emerged unpredictably during the recording period, occurring at least one hour after optogenetic stimulation and exhibiting no temporal relationship to the stimulus.

### Immunohistochemistry and RNA in situ hybridization

Mice were deeply anesthetized and transcardially perfused with phosphate-buffered saline (PBS) followed by 4% paraformaldehyde (PFA). Then, the brains were post-fixed in 4% PFA overnight at 4 °C and dehydrated with a 30% sucrose solution for 4 days. Coronal slices (40 μm) were obtained using a freezing microtome (Leica, CM1950, Germany) and stored in a freezing solution (2 × PBS: glycerol: ethylene glycol = 2:1:1) at −20 °C until use. The sections were washed three times with PBS and then incubated with a blocking solution (0.1% Triton-100/5% donkey serum in PBS) at room temperature. One hour later, the sections were moved to an incubation solution containing a primary rabbit anti-GABA (1:1000, Sigma-Aldrich, A2052), rabbit anti-glutamate (1:1000, Sigma-Aldrich, G6642), or guinea pig anti-Cre (1:500, SYSY, 257004) antibody and incubated overnight at 4 °C. The next day, the sections were washed four times with PBS and then incubated with a fluorescent secondary antibody solution containing Alexa Fluor 488, Alexa Fluor 594, Alexa Fluor 647 (1:500, Jackson ImmunoResearch), and DAPI (Sigma-Aldrich, 236276). The slides were imaged by a confocal microscope (AX, Nikon) or a fast-scan microscope (VS120, Olympus).

Fluorescence signals overlaid with DAPI or Nissl were considered positive cells. Immunolabeled cells were quantified using confocal images captured at 10× or 20×  magnification. During imaging, exposure time, laser intensity, and gain were kept consistent across all samples within the same experiment. Images were processed using FIJI (ImageJ) software, where a double-blind experimenter defined the target area. The positive cells within the outlined region were counted.

### fMRI data analysis

The fMRI data were converted to an NIFTI format, and the initial 10 volumes were removed. The datasets were then preprocessed by slice-timing correction using the 5th-order Lagrange polynomial interpolation and motion correction and employing the Fourier interpolation registration algorithm as implemented in the Analysis of Functional Neuroimages package (https://afni.nimh.nih.gov). Datasets with motion artifacts of head rotation > 0.15° or/and head translation > 0.1 mm were excluded. Spatial smoothing was performed using a Gaussian spatial kernel with a full-width at half-maximum of 0.5 mm.

The functional EPI images were aligned with the corresponding structural images using a 2D rigid body transformation (FMRIB Software Library). All structural images were co-registered to the representative image selected as the first reference. Then, they were all averaged to obtain a study-specific EPI template. The Allen Atlas was registered to the study-specific EPI template using linear and nonlinear transformation (Advanced Normalization Tools, ANTS). For seed-based rsfMRI analysis, the PC and lateral entorhinal cortex (Lent) seeds were carefully identified based on a combination of the Allen Atlas, virus-targeted field, and BOLD activations. The time series of each ROI was extracted by averaging signals across all voxels within the ROI. Resting-state EPI data were detrended and filtered between 0.01 and 0.1 Hz. Pearson correlation coefficients were computed to assess the temporal relationships between the time series from different ROIs for each run. Group-wise comparisons of the resting-state functional connectivity matrices were conducted by independent *t*-tests, with multiple comparison corrections using the false discovery rate (FDR) method. Clusters characterized by an edge-adjacency comprising fewer than 60 voxels were excluded. Statistical analysis was performed using RESTplus scripts (https://flask-restplus.readthedocs.io/en/stable), and statistical significance was defined at a threshold of *P* < 0.05.

### iDISCO+ brain-wide imaging and data analysis

Mice were sacrificed 1 hour after kindling. Fixed brains were incubated with a primary rabbit anti-c-Fos antibody (1:300, SYSY, 226008) and a secondary donkey anti-rabbit IgG (H + L) antibody 647 (1:300, Sigma-Aldrich, SAB4600177) for 10 days each. Cleared brains were scanned horizontally using a light-sheet microscope (LiToneXL, Light Innovation Technology, China) with a 4 × objective lens (NA = 0.28, working distance = 20 mm), 2.9 μm × 2.9 μm resolution, and a 4-μm step.

The iDISCO+ staining protocol from http://www.idisco.info was modified. Mice were sacrificed one hour after kindling for c-Fos-iDISCO+. Brain-wide iDISCO+ datasets were analyzed using open-source ClearMap2 [76]. All datasets were registered to the Allen Institute’s Common Coordinate Framework through the Elastix toolbox [77]. To increase the accuracy of registration, all datasets were cropped to the approximate range of Bregma +2.34 mm to −4.36 mm, resampled from a resolution of 2.9 × 2.9 × 4 μm^3^ to 20 × 20 × 20 μm^3^, and registered to the Allen Institute’s Common Coordinate Framework through the Elastix toolbox. For cell detection, a background correction and maxima detection were performed. Quantitative analysis was conducted to assess the cell counts within the top 100 brain regions.

### Cross-analysis of iDISCO+ data and fMRI data

To match the MRI study, the atlas was registered to the study-specific EPI template using the same ANTS transformation matrix. Cell maps were then transformed using the same transformation matrix. Similar parameters were used for spatial smoothing and group-wise comparisons as in the MRI analysis. Statistical significance, defined at a threshold of *P* < 0.005, was selected for statistical assessments. To evaluate the spatial consistency of the BOLD-fMRI map and c-Fos map, the Dice coefficient was calculated using the following formula [78]:


$$\text{Dice}\;(X,\;Y)=\;\frac{\text{2}\;*\;|\;X\cap \;Y\;|}{\left|\;X\;|+|Y\;\right|},$$

where *X* and *Y* represent the number of voxels with c-Fos signals (*t* test, FDR-corrected, *P* < 0.005) and BOLD-fMRI sustained responses (*t t*est, FDR-corrected, *P* < 0.005 between the baseline and the mean of the last 15 volumes), respectively, and *X* ∩ *Y* is the number of voxels exhibiting co-activation. Its values range between 0 and 1, where 0 indicates no overlap between the two sets, while 1 signifies a complete overlap between the two sets.

### Clinical imaging analysis

The generated data were used for PC segmentation references and volumetric analysis. The delineation of the PC was based on histologically defined anatomical landmarks [79] and the steps proposed by Galovic and colleagues [40]. Notably, according to previous studies, the PC and periamygdaloid cortex area are spatially and functionally connected in the human brain and could not be discriminated in MRI. We manually outlined the PC ROI, as described previously [39,40]. The PC segmentation was performed by a radiologist (J.Z.) using the MRIcron software and then carefully reviewed and revised by a senior expert radiologist (N.W.) after a 2-week group discussion and training with other authors. The manual segmentation of the MRI data was performed by clinicians who were not blinded to the patient and control groups. Specifically, the approximate location of the PC was identified by displaying each image in multiple planes and using free-surfer-generated neighbor regions as references. The ROIs were mainly outlined in coronal slices and modified in other planes. The last outlined slice was selected based on the appearance of the mammillary bodies. The mean and SD of each ROI’s volume were calculated for all groups.

3D T1 images were rotated to align with the anterior and posterior commissures. Then, a “recon-all” reconstruction analysis was performed on each image, generating segmentation results of standard brain regions, involving the hippocampus, amygdala, and entorhinal cortex, as well as an estimated total intracranial volume. To assess the degree of volume decrease for each comparison, the volume change ratio was calculated as the percentage of mean volume change in the patient group compared with the corresponding volume in controls, expressed as (control−patient)/control × 100%.

## Statistical analysis

The data were analyzed using MATLAB 2021b (MathWorks, USA) and GraphPad Prism 8.0.2 (GraphPad Software, USA). The data are presented as mean ± standard error of the mean (SEM) and whisker: min–max values, box: 25th–75th percentile. Shapiro–Wilk normality tests were used to estimate whether the values conform to a Gaussian distribution. If the data followed a normal distribution, statistical analyses were performed using a two-tailed *t* test, unpaired *t* test (or unpaired *t* test with Welch’s correction), one-way analysis of variance (ANOVA) followed by Tukey’s multiple comparison test (or Dunnett’s multiple comparisons test), or two-way repeated measures ANOVA followed by Bonferroni’s multiple comparison test. Otherwise, the Mann–Whitney test was used for unpaired two-group comparisons, and the Wilcoxon signed-rank test was used for paired two-group comparisons. The Pearson correlation coefficient was used to assess the correlation between two continuous variables. *P* value < 0.05 was considered statistically significant. NS, not significant.

## Supporting information

S1 Fig Volumetric comparisons between patients with TLE and healthy controls. (**A–C**) Volume changes in the Ent, the Amy, and the Hip (Control versus lTLE: unpaired *t* test, *t*_40_ = 7.624, *P* < 0.0001) of patients with TLE relative to healthy controls. ****P < 0.0001. The data underlying this Figure can be found in S1 Data.
(TIF)

S2 Fig The number of glutamatergic neurons in the PC. (**A**) Left, a representative image shows the expression of *Vglut1* and *Vglut2* in the PC; scale bar: 200 μm. Right, the enlarged image from the left dashed square; scale bar: 50 μm. (**B**) The number of *Vglut1*^+^ (1,133 ± 142.2 cells/mm^2^; *n* = 4 mice) and *Vglut2*^+^ neurons (22.44 ± 0.8107 cells/mm^2^; *n* = 4 mice) in the PC (unpaired *t* test with Welch’s correction, *t* = 7.810, *P* = 0.0044, *n* = 4). (**C**) MERFISH spatial transcriptomic analysis of the co-localization of *CaMKIIα* and *Vglut1* in PC (from Allen Brain, C57BL6J-63885, https://knowledge.brain-map.org/abcatlas). (D) *CaMKIIα* and *Vglut1* co-expression in the PC. *Vglut1*: vesicular glutamate transporter 1; *Vglut2*: vesicular glutamate transporter 2. **P < 0.01. The data underlying this Figure can be found in S1 Data.
(TIF)

S3 Fig Opto-kindling of vglut1 neurons in the PC induces anxiety-like behavior. (**A**) Representative heatmaps of time spent in the EPM test. (**B**) The time spent in the open arms during the EPM test (Control versus Kindled, Unpaired *t* test, *t*_24_ = 3.447, *P* = 0.0021). (**C**) The number of entries to open arms (Control versus Kindled, Unpaired *t* test, *t*_24_ = 3.933, *P* = 0.0006). (**D**) Movement distance in the open arms (Control versus Kindled, Unpaired *t* test, *t*_24_ = 3.956, *P* = 0.0006). (**E**) Locomotor activity in the EPM test (Control versus Kindled, Unpaired *t* test, *t*_24_ = 0.9887, *P* = 0.3327). *n* = 14 for the control group and 12 for the kindled group. NS, not significant, **P < 0.01, ***P < 0.001. EPM: elevated-plus-maze. The data underlying this Figure can be found in S1 Data.
(TIF)

S4 Fig Optogenetic and chemogenetic manipulations of vglut1 neurons in the PC. (**A**) The experimental scheme of the optogenetic viral injection and behavioral test. (**B**) Left, an averaged time course of Ca^2+^ signals (∆*F*/*F*) with different opto-powers. Right, the peak Ca^2+^ signal (one-way ANOVA followed by Tukey’s multiple comparison test, 0.3 mW versus 1 mW, *P* = 0.0019; 1 mW versus 3 mW, *P* = 0.6622; *n* = 12). (**C**) Representative LFP (top) and EEG (bottom) signals from the same mouse during stimulation with different opto-powers. Red bar: 10-s light stimulation. (**D**) The number of seizures induced by different opto-powers (one-way ANOVA followed by Tukey’s multiple comparison test, 0.3 mW versus 1 mW, *P* = 0.001; 1 mW versus 3 mW, *P* = 0.034; *n* = 12). (**E**) Top left, the experimental scheme of chemogenetic viral injection, electrophysiological recording, and calcium signal recording. Top right, the representative image shows mixed viral expression in the PC; scale bar: 200 μm. Bottom, enlarged images show AAV-DIO-hM3Dq-mCherry and AAV-hSyn-GCaMP6m expression in the PC; scale bars: 50 μm. (**F**) Representative LFP (top) and EEG traces (bottom) from the same mouse during CNO administration. The artifact indicates the time point of CNO injection. (**G**) Typical calcium signals of an FS (left) and GS (right) from one mouse after CNO administration. *P < 0.05, **P < 0.01. FS: focal seizure; GS: generalized seizure; *Vglut1*: vesicular glutamate transporter 1. The data underlying this Figure can be found in S1 Data.
(TIF)

S5 Fig SRSs evoked by rekindling in PC-kindled mice. (**A**) The experimental scheme of the viral injection and EEG recording. (**B**) Left, the rekindling timeline. Right, the image shows the 24-hour EEG monitoring device. (**C**) Representative EEG traces accompanied by SRSs in a mouse. (**D**) An enlarged EEG trace from (C) and the corresponding power spectrogram of the EEG signal. (**E**) The number of mice exhibiting SRSs. (**F**) The number of SRSs during the first 24 hour from a representative mouse. (**G**) The number of SRSs in each mouse (*n* = 11). (**H**) The average number of SRSs per day at the early stage (first 3 days) and later stage (last 3 days) (Wilcoxon signed-rank test, *P* = 0.0039, *n* = 11). **P < 0.01. PC: piriform cortex; SRSs: spontaneous recurrent seizures. The data underlying this Figure can be found in S1 Data.
(TIF)

S6 Fig ClearMap analysis shows activations in PC-kindled mice. (**A**) Left, c-Fos iDISCO+ brain staining. Right, representative image of c-Fos expression. Bottom, automatic detection of ClearMap’s c-Fos signal; scale bars: 1 mm. (**B**) Fluorescence mapping in a cleared brain imaged after c-Fos-iDISCO+ staining. Left, a control mouse brain. Right, a PC-kindled mouse brain. Dorsal (D), medial (M), and caudal (C) directions are denoted; scale bars: 1 mm. (**C**) c-Fos expression after opto-kindling in networks inducing: midbrain (MID), hypothalamus (HYPO), thalamus (TH), forebrain nuclei (FN), cortical subplate (CS), hippocampus (HPC), olfactory (OLF), and isocortex (CTX). (**D**) Top, comparison of activation between ChR2- and GFP-injected mice. Red blobs indicate voxels with significant differences (ChR2 > GFP, *P* < 0.05, *n* = 6). Bottom, spatial consistency of BOLD (*P* < 0.005, *n* = 20) and c-Fos maps (*P* < 0.005, *n* = 6). OB: olfactory bulb; SN: substantia nigra; VTA: ventral tegmental area; MRN: midbrain reticular nucleus; PG: periaqueductal gray; MB: midbrain; VHN: ventromedial hypothalamic nucleus; AHN: arcuate hypothalamic nucleus; MPN: medial preoptic nucleus; TN: tuberal nucleus; RA: retrochiasmatic area; Rt: reticular nucleus of the thalamus; LPT: lateral posterior nucleus of the thalamus; SN: subparafascicular nucleus; MG: medial geniculate complex; PN: peripeduncular nucleus; MA: medial amygdalar nucleus; BNAO: bed nucleus of the accessory olfactory tract; BNST: bed nuclei of the stria terminalis; SAN: striatum-like amygdalar nuclei; CeA: central amygdalar nucleus; PA: posterior amygdalar nucleus; BMA: basomedial amygdalar nucleus; BLA: basolateral amygdalar nucleus; LA: lateral amygdalar nucleus; CS: cortical subplate; Sub: subiculum; Et: entorhinal area; PPT: postpiriform transition area; CA: cortical amygdalar area; PA: piriform-amygdalar area; LOT: nucleus of the lateral olfactory tract; PC: piriform area; VA: visceral area; SSA: supplemental somatosensory area; GA: gustatory areas; PM: primary motor area; Prh: perirhinal area. The data underlying this Figure can be found in S1 Data.
(TIF)

S7 Fig Normalized number of GFP^+^ cells in each region. The data underlying this Figure can be found in S1 Data.
(TIF)

S8 Fig PC neurons project to the bilateral Lent. (**A**) Left, the expression of Cre and ChrimsonR in the ipsilateral PC; scale bar: 100 μm. Right, enlarged images from the left dashed square; scale bars: 40 μm. (B) Left, Cre, and Cre-GFP expression in the contralateral PC; scale bar: 100 μm. Right, enlarged images from the left dashed square; scale bars: 10 μm. (**C**) Left, scAAV-flex-GFP was injected into the unilateral PC. Right, representative image showing GFP^+^ cells in the bilateral Lent. Scale bar: 1 mm. (**D**) High-magnification view of GFP^+^ cells in the contralateral Lent. Scale bar: 100 μm.
(TIF)

S9 Fig Positive neurons in two hemispheres by anterograde tracing and c-Fos mapping. (**A**) Quantification of PC-projecting neurons in the contralateral hemisphere. (**B**) c-Fos expression in the ipsilateral and contralateral hemispheres following PC-kindling-induced seizures. The data underlying this Figure can be found in S1 Data.
(TIF)

S10 Fig Suppressing the direct and indirect downstream brain regions of the PC. (**A**) Inhibition of the bilateral PC-PRh circuit using TetTox. Scale bar: 200 μm. (**B**) Inhibition of the bilateral PC-BLA circuit with TetTox. Scale bar: 200 μm. (**C**) Suppressing the bilateral motor cortex. Scale bar: 200 μm. (**D**) Suppressing the bilateral RSC. Scale bar: 200 μm. (**E**) Daily number of GSs, FSs, and total seizures; *n* = 6 from each group. (**F**) Comparison of the seizure number (one-way ANOVA followed by Dunnett’s multiple comparisons test, Lent versus Prh, *P* = 0.0216; Lent versus BLA, *P* = 0.0070; Lent versus M1, *P* < 0.0001; Lent versus RSC, *P* < 0.0001). *P < 0.05, **P < 0.01, ****P < 0.0001. The data underlying this Figure can be found in S1 Data.
(TIF)

S11 Fig PC projects predominantly to glutaminergic neurons in the Lent. (**A**) Top, Glut^+^ and mCherry^+^ cells. Bottom, GABA^+^ and mCherry^+^ cells. Scale bars: 50 μm and 20 μm (enlarged images). (**B**) Percentage of mCherry^+^ cells co-labeled with Glut or GABA (Mann–Whitney test, *P* < 0.0001, *n* = 10). ****P < 0.0001. GABA: gamma-aminobutyric acid; Glut: glutamate; Lent: lateral entorhinal cortex. The data underlying this Figure can be found in S1 Data.
(TIF)

S12 Fig Overlapping of PC-anterograde tracing and DG-retrograde tracing in the Lent. (**A**) Images show that CTB-647 and CTB-555 were injected into the dDG (left) and vDG (right), respectively; scale bars: 200 μm. (**B**) Left, the image shows GFP^+^ neurons co-localization with vDG-projection cells (red, CTB-555) and dDG-projection cells (white, CTB-647) in the Lent; scale bar: 100 μm. Right, an enlarged image from layers II–III of the Lent; scale bars: 10 μm. Yellow arrows indicate overlapping cells. (**C**) Number of GFP^+^ cells from the PC in layers II–III of the Lent projecting to the dDG (CTB-647^+^) and vDG (CTB-555^+^). Bregma −2.2 mm: vDG (4.800 ± 1.800); dDG (3.600 ± 1.568); Bregma −2.8 mm: vDG (17.00 ± 1.140); dDG (13.20 ± 1.625); Bregma −3.1 mm: vDG (22.20 ± 3.153); dDG (17.60 ± 3.076); Bregma −3.6 mm: vDG (23.40 ± 3.614); dDG (17.40 ± 3.473); Bregma −4.0 mm: vDG (26.60 ± 3.311); dDG (16.80 ± 4.128), *n* = 5. PC: piriform cortex; DG: dentate gyrus; dDG: dorsal dentate gyrus; Lent: lateral entorhinal cortex; vDG: ventral dentate gyrus. The data underlying this Figure can be found in S1 Data.
(TIF)

S13 Fig Dynamics of Ca^2+^ signaling in the PC, the Lent, and the DG during kindling. (**A**) Representative calcium recordings from a mouse across four consecutive days of kindling. (**B**) Duration of calcium response in the PC (Day 1 versus Day 4, *P* = 0.0130), the Lent (Day 1 versus Day 4, *P* < 0.0001; Day 1 versus Day 3, *P* = 0.0071; Day 2 versus Day 4, *P* = 0.0007), and the DG (Day 1 versus Day 4, *P* = 0.0153) over the kindling period. Repeated measures one-way ANOVA followed by Tukey’s post hoc test, *n* = 6. *P < 0.05, **P < 0.01, ***P < 0.001, ****P < 0.0001. The data underlying this Figure can be found in S1 Data.
(TIF)

S1 Movie A spontaneous seizure following the rekindling procedure. A spontaneous seizure occurred 5 days after the second stimulation of a kindled mouse.
(MP4)

S2 Movie A PC^Vglut1^-kindling-induced generalized seizure in an awake mouse. Left, a generalized seizure occurred following opto-kindling in the PC. Right, no changes were found in the control mouse. A 10-s, 20 Hz optical stimulation with a pulse width of 5 ms begins at 5 s in the movie.
(MP4)

S1 Data Numerical data related to the main figures and supporting figures. (XLSX)

## Abbreviations

ANOVA: analysis of variance
ANTS: advanced Normalization Tools
BLA: basolateral amygdaloid nucleus
BOLD: blood-oxygen-level-dependent
ChR2: channelrhodopsin-2
DG: dentate gyrus
EEGs: electroencephalograms
EPI: echo planar imaging
EPM: elevated-plus-maze
FDR: false discovery rate
FOV: field of view
GSs: generalized seizures
Lent: lateral entorhinal cortex
LFPs: local field potentials
mPFC: medial prefrontal cortex
nLot: nucleus of the lateral olfactory tract
ofMRI: optogenetic functional MRI
PBS: phosphate-buffered saline
PFA: paraformaldehyde
PC: piriform cortex
PRh: perirhinal cortex
rsFC: resting-state functional connectivity
rsfMRI: resting-state fMRI
SRSs: spontaneous recurrent seizures
SuM: supramammillary nucleus
TLE: temporal lobe epilepsy
