# Supplementary figures and images for: Piriform seizures mediated by the piriform-entorhino-dentate circuit induce brain-wide functional reorganization in mice
Source: PLoS Biol. 2026 Feb 12;24(2):e3003577. doi: 10.1371/journal.pbio.3003577 (PMC12900355; doi:10.1371/journal.pbio.3003577)

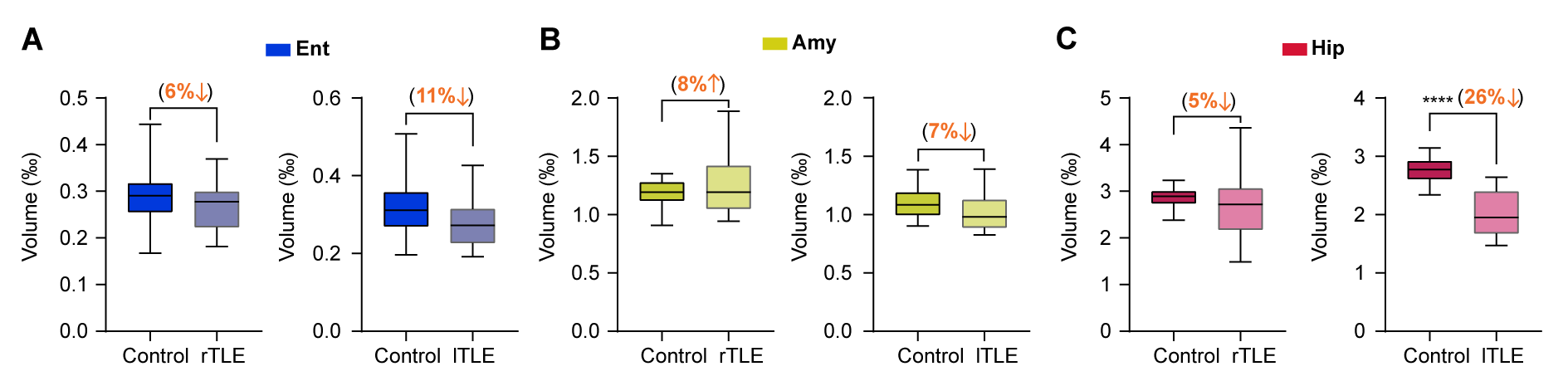

Supplement: S1 Fig — (A–C) Volume changes in the Ent, the Amy, and the Hip (Control versus lTLE: unpaired t test, t40 = 7.624, P < 0.0001) of patients with TLE relative to healthy controls. ****P < 0.0001. The data underlying this Figure can be found in S1 Data. (TIF) [file pbio.3003577.s001.tif]

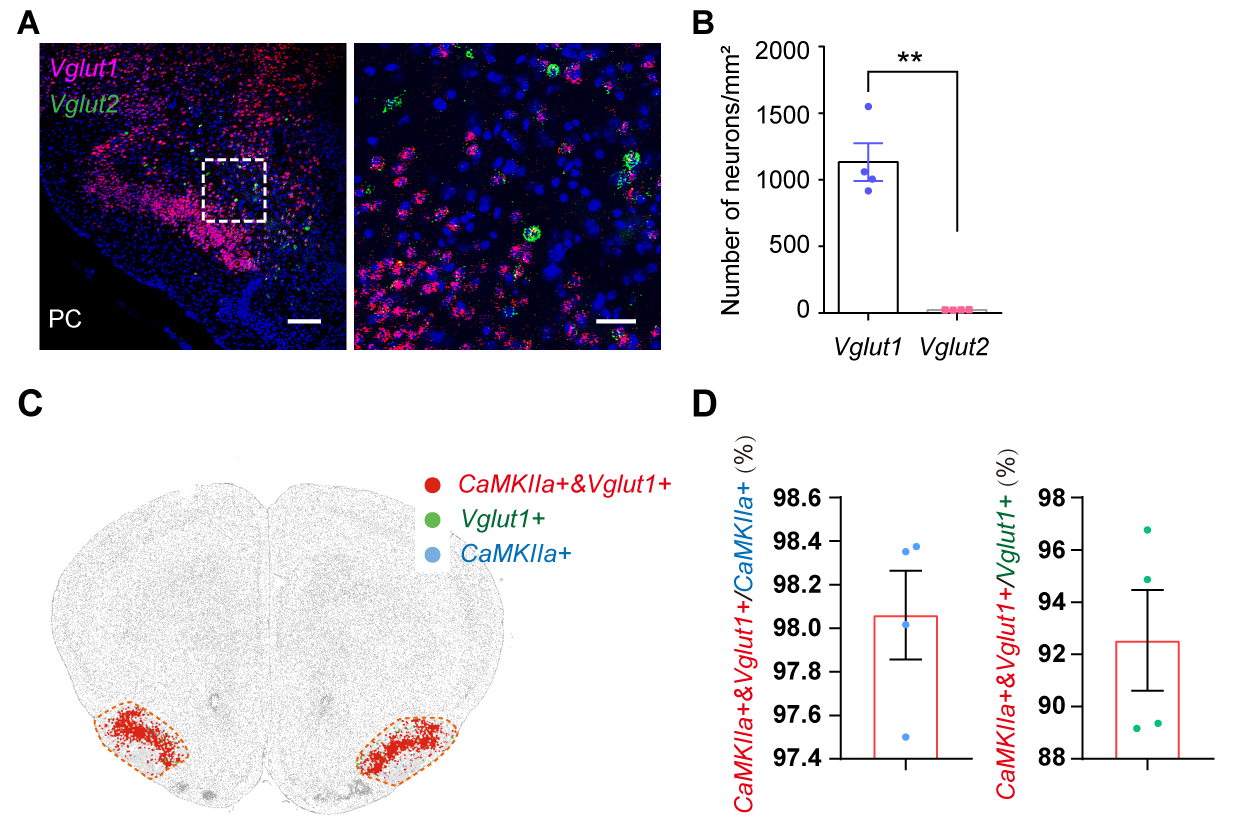

Supplement: S2 Fig — (A) Left, a representative image shows the expression of Vglut1 and Vglut2 in the PC; scale bar: 200 μm. Right, the enlarged image from the left dashed square; scale bar: 50 μm. (B) The number of Vglut1+ (1,133 ± 142.2 cells/mm2; n = 4 mice) and Vglut2+ neurons (22.44 ± 0.8107 cells/mm2; n = 4 mice) in the PC (unpaired t test with Welch’s correction, t = 7.810, P = 0.0044, n = 4). (C) MERFISH spatial transcriptomic analysis of the co-localization of CaMKIIα and Vglut1 in PC (from Allen Brain, C57BL6J-63885, https://knowledge.brain-map.org/abcatlas). (D) CaMKIIα and Vglut1 co-expression in the PC. Vglut1: vesicular glutamate transporter 1; Vglut2: vesicular glutamate transporter 2. **P < 0.01. The data underlying this Figure can be found in S1 Data. (TIF) [file pbio.3003577.s002.tif]

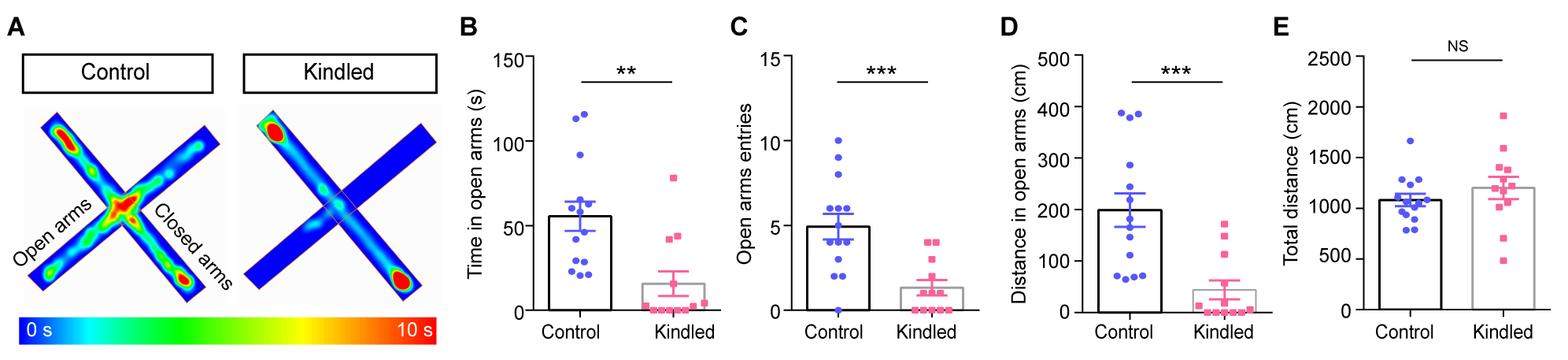

Supplement: S3 Fig — (A) Representative heatmaps of time spent in the EPM test. (B) The time spent in the open arms during the EPM test (Control versus Kindled, Unpaired t test, t24 = 3.447, P = 0.0021). (C) The number of entries to open arms (Control versus Kindled, Unpaired t test, t24 = 3.933, P = 0.0006). (D) Movement distance in the open arms (Control versus Kindled, Unpaired t test, t24 = 3.956, P = 0.0006). (E) Locomotor activity in the EPM test (Control versus Kindled, Unpaired t test, t24 = 0.9887, P = 0.3327). n = 14 for the control group and 12 for the kindled group. NS, not significant, **P < 0.01, ***P < 0.001. EPM: elevated-plus-maze. The data underlying this Figure can be found in S1 Data. (TIF) [file pbio.3003577.s003.tif]

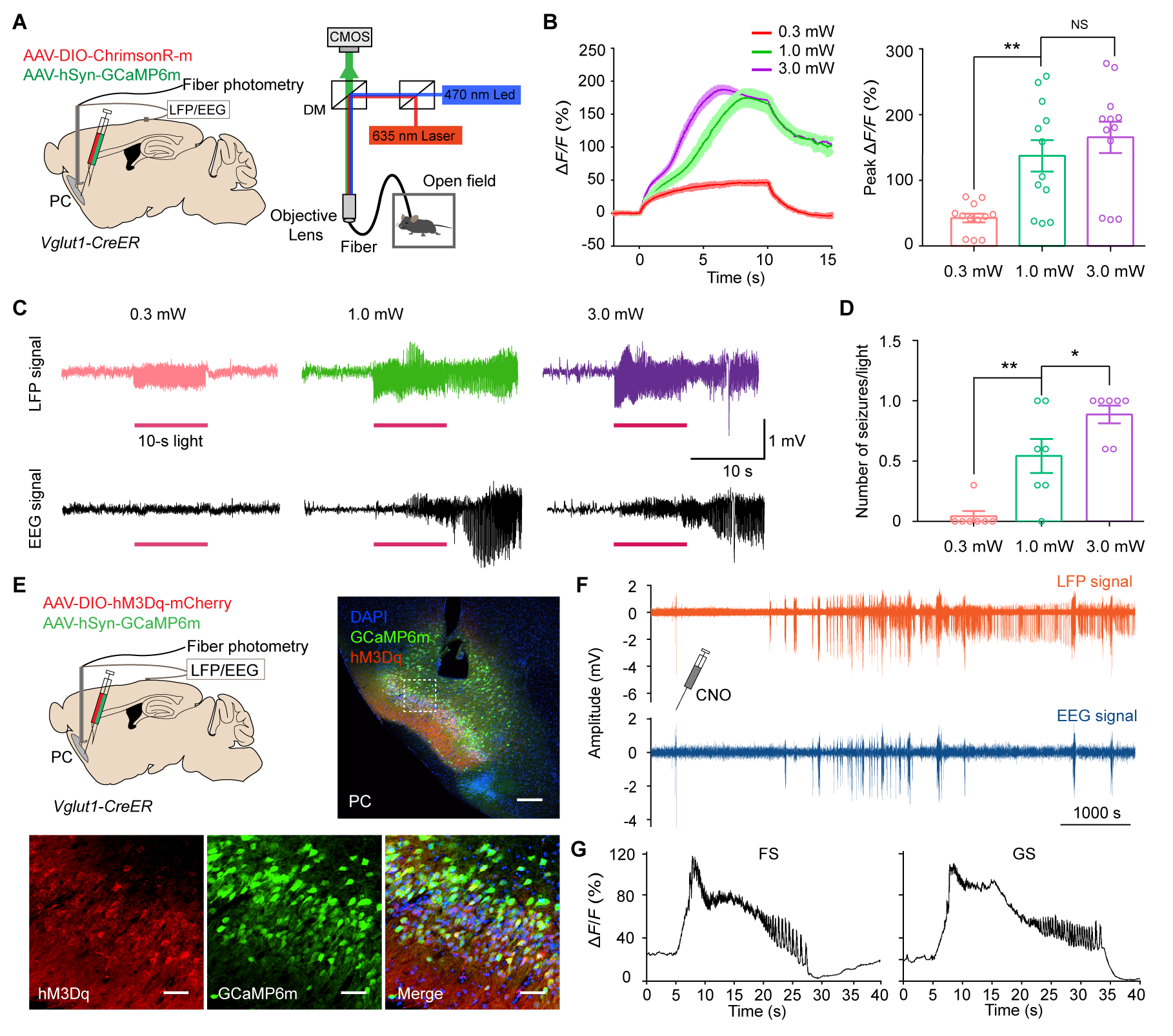

Supplement: S4 Fig — (A) The experimental scheme of the optogenetic viral injection and behavioral test. (B) Left, an averaged time course of Ca2+ signals (∆F/F) with different opto-powers. Right, the peak Ca2+ signal (one-way ANOVA followed by Tukey’s multiple comparison test, 0.3 mW versus 1 mW, P = 0.0019; 1 mW versus 3 mW, P = 0.6622; n = 12). (C) Representative LFP (top) and EEG (bottom) signals from the same mouse during stimulation with different opto-powers. Red bar: 10-s light stimulation. (D) The number of seizures induced by different opto-powers (one-way ANOVA followed by Tukey’s multiple comparison test, 0.3 mW versus 1 mW, P = 0.001; 1 mW versus 3 mW, P = 0.034; n = 12). (E) Top left, the experimental scheme of chemogenetic viral injection, electrophysiological recording, and calcium signal recording. Top right, the representative image shows mixed viral expression in the PC; scale bar: 200 μm. Bottom, enlarged images show AAV-DIO-hM3Dq-mCherry and AAV-hSyn-GCaMP6m expression in the PC; scale bars: 50 μm. (F) Representative LFP (top) and EEG traces (bottom) from the same mouse during CNO administration. The artifact indicates the time point of CNO injection. (G) Typical calcium signals of an FS (left) and GS (right) from one mouse after CNO administration. *P < 0.05, **P < 0.01. FS: focal seizure; GS: generalized seizure; Vglut1: vesicular glutamate transporter 1. The data underlying this Figure can be found in S1 Data. (TIF) [file pbio.3003577.s004.tif]

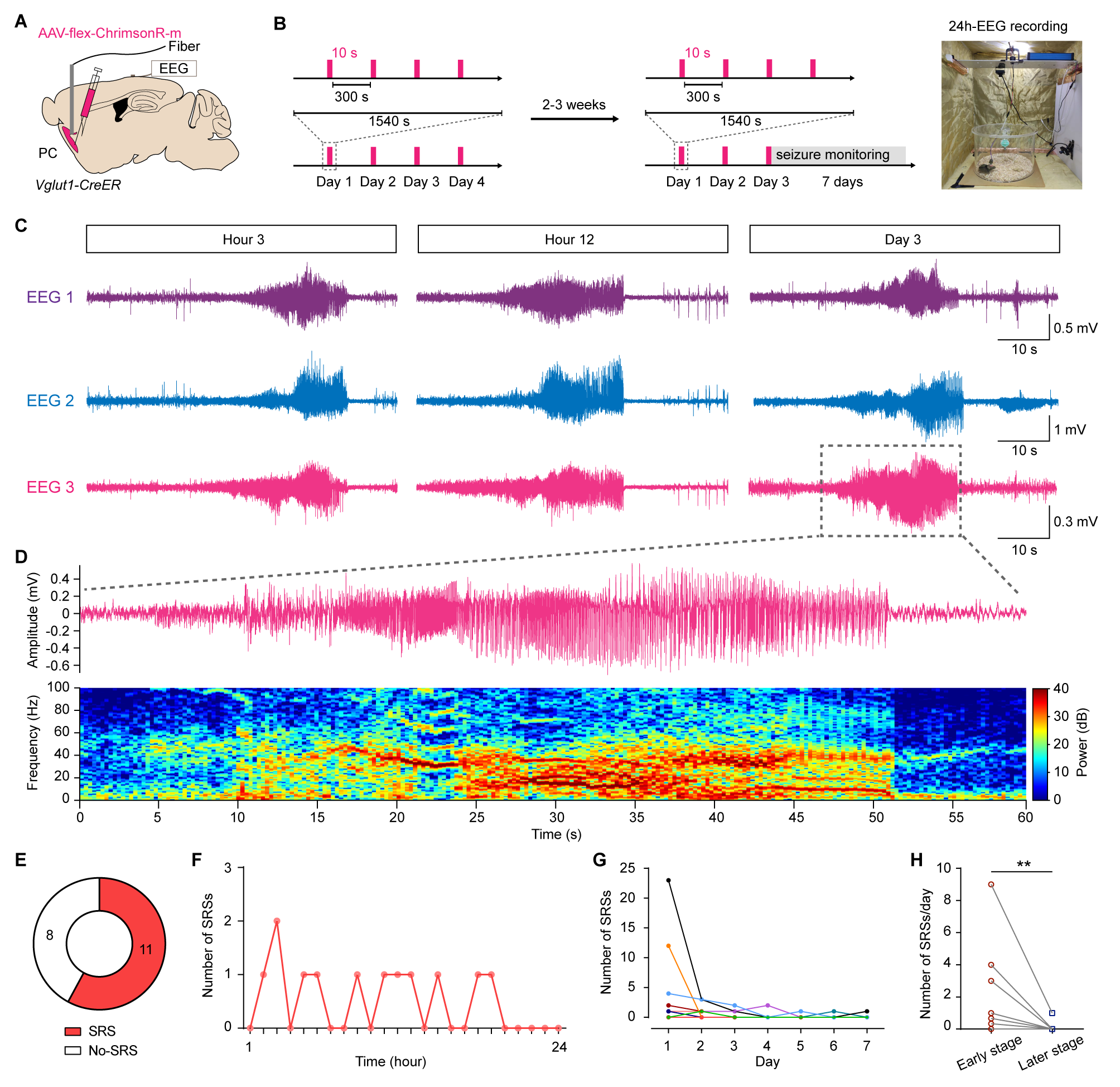

Supplement: S5 Fig — (A) The experimental scheme of the viral injection and EEG recording. (B) Left, the rekindling timeline. Right, the image shows the 24-hour EEG monitoring device. (C) Representative EEG traces accompanied by SRSs in a mouse. (D) An enlarged EEG trace from (C) and the corresponding power spectrogram of the EEG signal. (E) The number of mice exhibiting SRSs. (F) The number of SRSs during the first 24 hour from a representative mouse. (G) The number of SRSs in each mouse (n = 11). (H) The average number of SRSs per day at the early stage (first 3 days) and later stage (last 3 days) (Wilcoxon signed-rank test, P = 0.0039, n = 11). **P < 0.01. PC: piriform cortex; SRSs: spontaneous recurrent seizures. The data underlying this Figure can be found in S1 Data. (TIF) [file pbio.3003577.s005.tif]

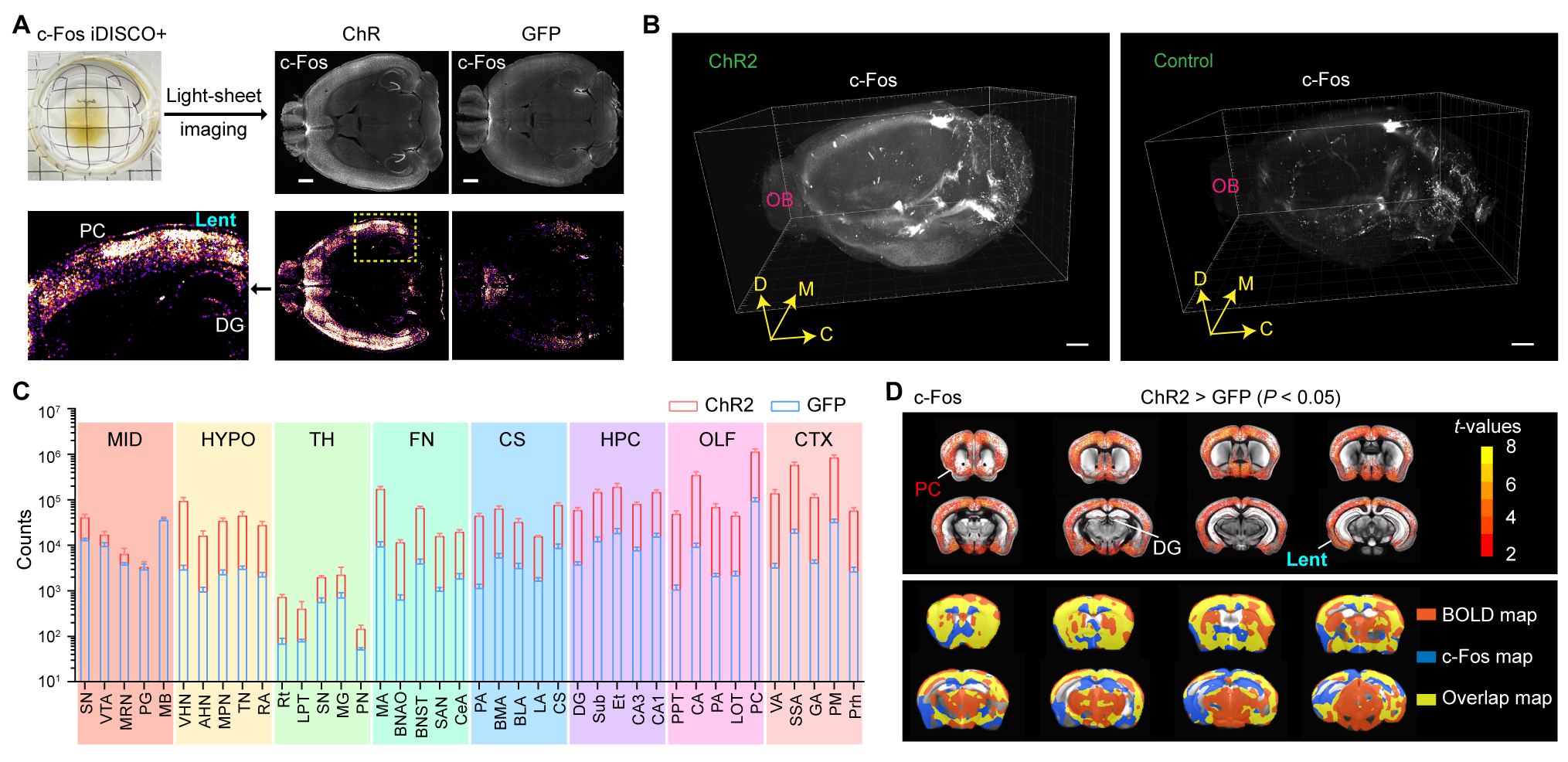

Supplement: S6 Fig — (A) Left, c-Fos iDISCO+ brain staining. Right, representative image of c-Fos expression. Bottom, automatic detection of ClearMap’s c-Fos signal; scale bars: 1 mm. (B) Fluorescence mapping in a cleared brain imaged after c-Fos-iDISCO+ staining. Left, a control mouse brain. Right, a PC-kindled mouse brain. Dorsal (D), medial (M), and caudal (C) directions are denoted; scale bars: 1 mm. (C) c-Fos expression after opto-kindling in networks inducing: midbrain (MID), hypothalamus (HYPO), thalamus (TH), forebrain nuclei (FN), cortical subplate (CS), hippocampus (HPC), olfactory (OLF), and isocortex (CTX). (D) Top, comparison of activation between ChR2- and GFP-injected mice. Red blobs indicate voxels with significant differences (ChR2 > GFP, P < 0.05, n = 6). Bottom, spatial consistency of BOLD (P < 0.005, n = 20) and c-Fos maps (P < 0.005, n = 6). OB: olfactory bulb; SN: substantia nigra; VTA: ventral tegmental area; MRN: midbrain reticular nucleus; PG: periaqueductal gray; MB: midbrain; VHN: ventromedial hypothalamic nucleus; AHN: arcuate hypothalamic nucleus; MPN: medial preoptic nucleus; TN: tuberal nucleus; RA: retrochiasmatic area; Rt: reticular nucleus of the thalamus; LPT: lateral posterior nucleus of the thalamus; SN: subparafascicular nucleus; MG: medial geniculate complex; PN: peripeduncular nucleus; MA: medial amygdalar nucleus; BNAO: bed nucleus of the accessory olfactory tract; BNST: bed nuclei of the stria terminalis; SAN: striatum-like amygdalar nuclei; CeA: central amygdalar nucleus; PA: posterior amygdalar nucleus; BMA: basomedial amygdalar nucleus; BLA: basolateral amygdalar nucleus; LA: lateral amygdalar nucleus; CS: cortical subplate; Sub: subiculum; Et: entorhinal area; PPT: postpiriform transition area; CA: cortical amygdalar area; PA: piriform-amygdalar area; LOT: nucleus of the lateral olfactory tract; PC: piriform area; VA: visceral area; SSA: supplemental somatosensory area; GA: gustatory areas; PM: primary motor area; Prh: perirhinal area. The d [file pbio.3003577.s006.tif]

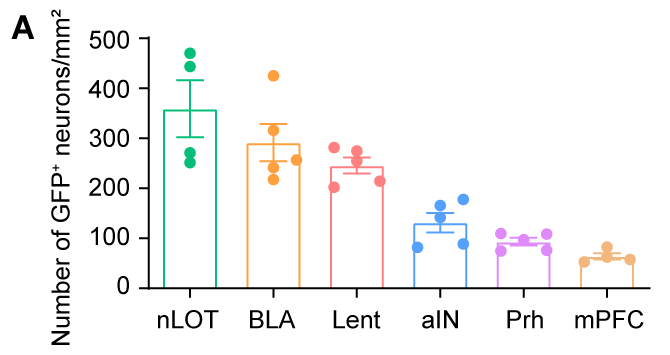

Supplement: S7 Fig — The data underlying this Figure can be found in S1 Data. (TIF) [file pbio.3003577.s007.tif]

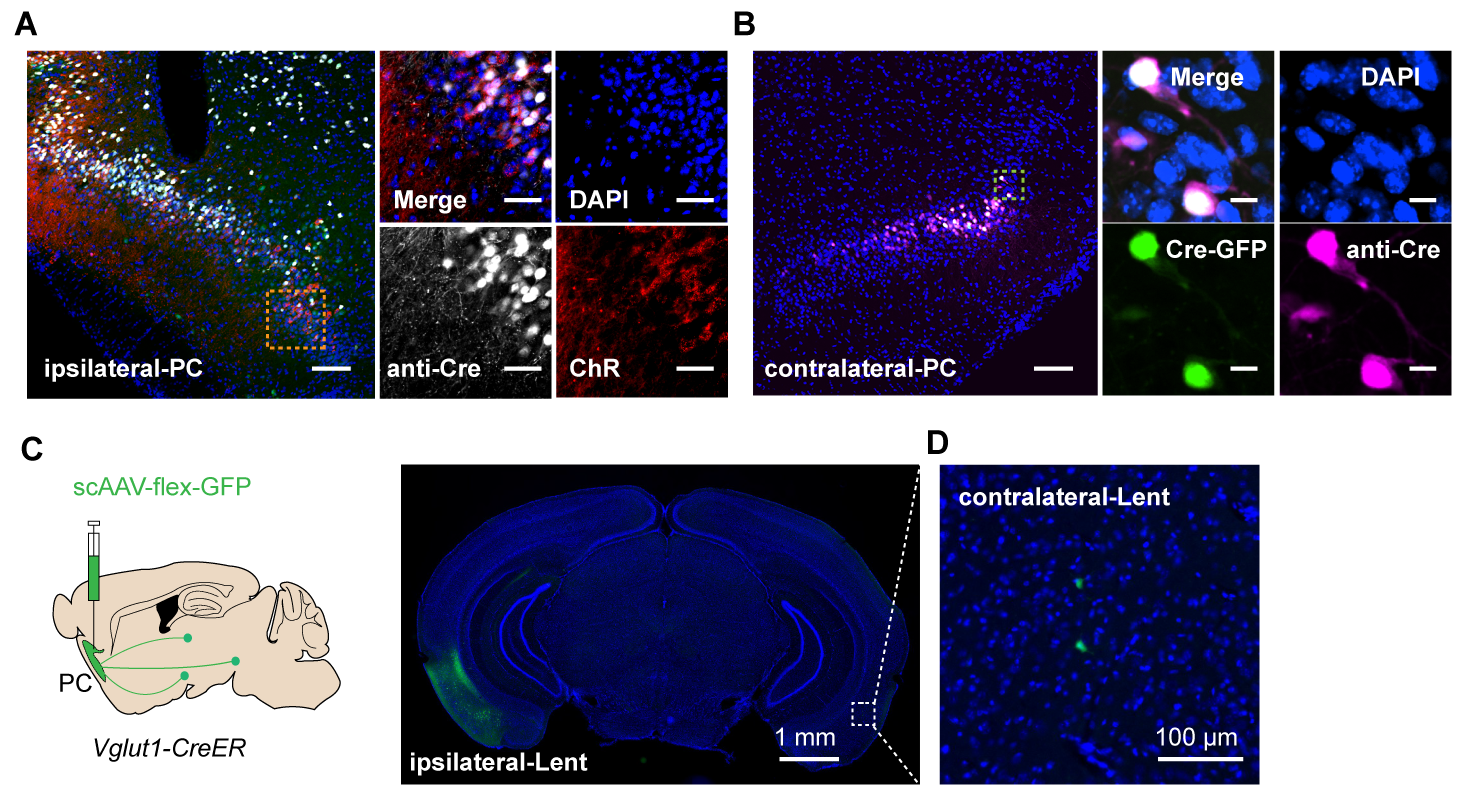

Supplement: S8 Fig — (A) Left, the expression of Cre and ChrimsonR in the ipsilateral PC; scale bar: 100 μm. Right, enlarged images from the left dashed square; scale bars: 40 μm. (B) Left, Cre, and Cre-GFP expression in the contralateral PC; scale bar: 100 μm. Right, enlarged images from the left dashed square; scale bars: 10 μm. (C) Left, scAAV-flex-GFP was injected into the unilateral PC. Right, representative image showing GFP+ cells in the bilateral Lent. Scale bar: 1 mm. (D) High-magnification view of GFP+ cells in the contralateral Lent. Scale bar: 100 μm. (TIF) [file pbio.3003577.s008.tif]

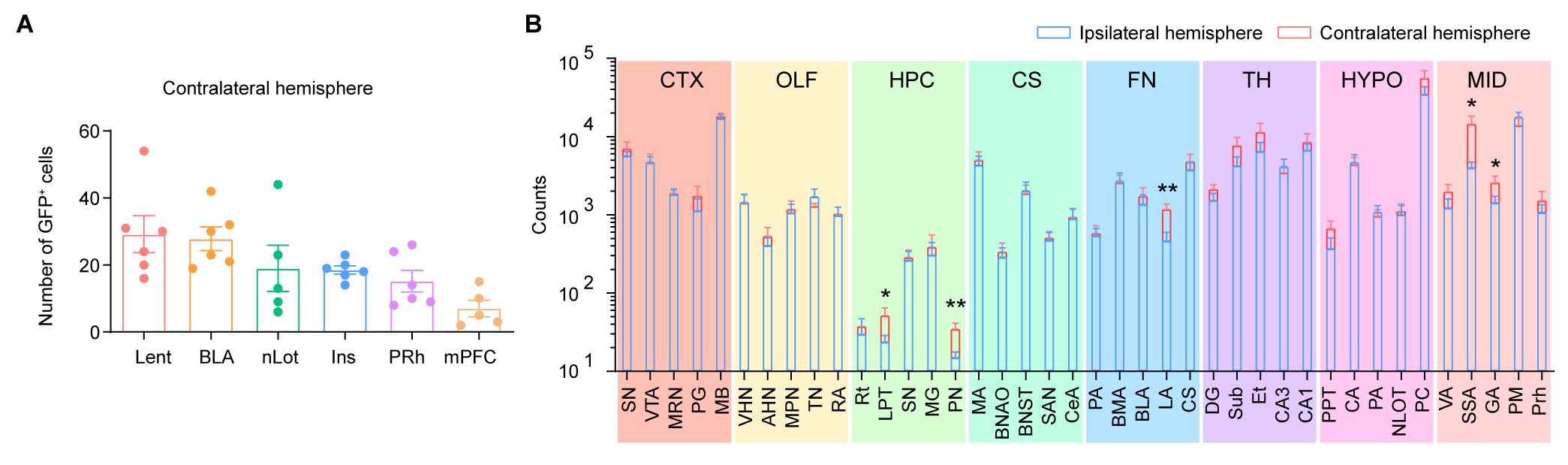

Supplement: S9 Fig — (A) Quantification of PC-projecting neurons in the contralateral hemisphere. (B) c-Fos expression in the ipsilateral and contralateral hemispheres following PC-kindling-induced seizures. The data underlying this Figure can be found in S1 Data. (TIF) [file pbio.3003577.s009.tif]

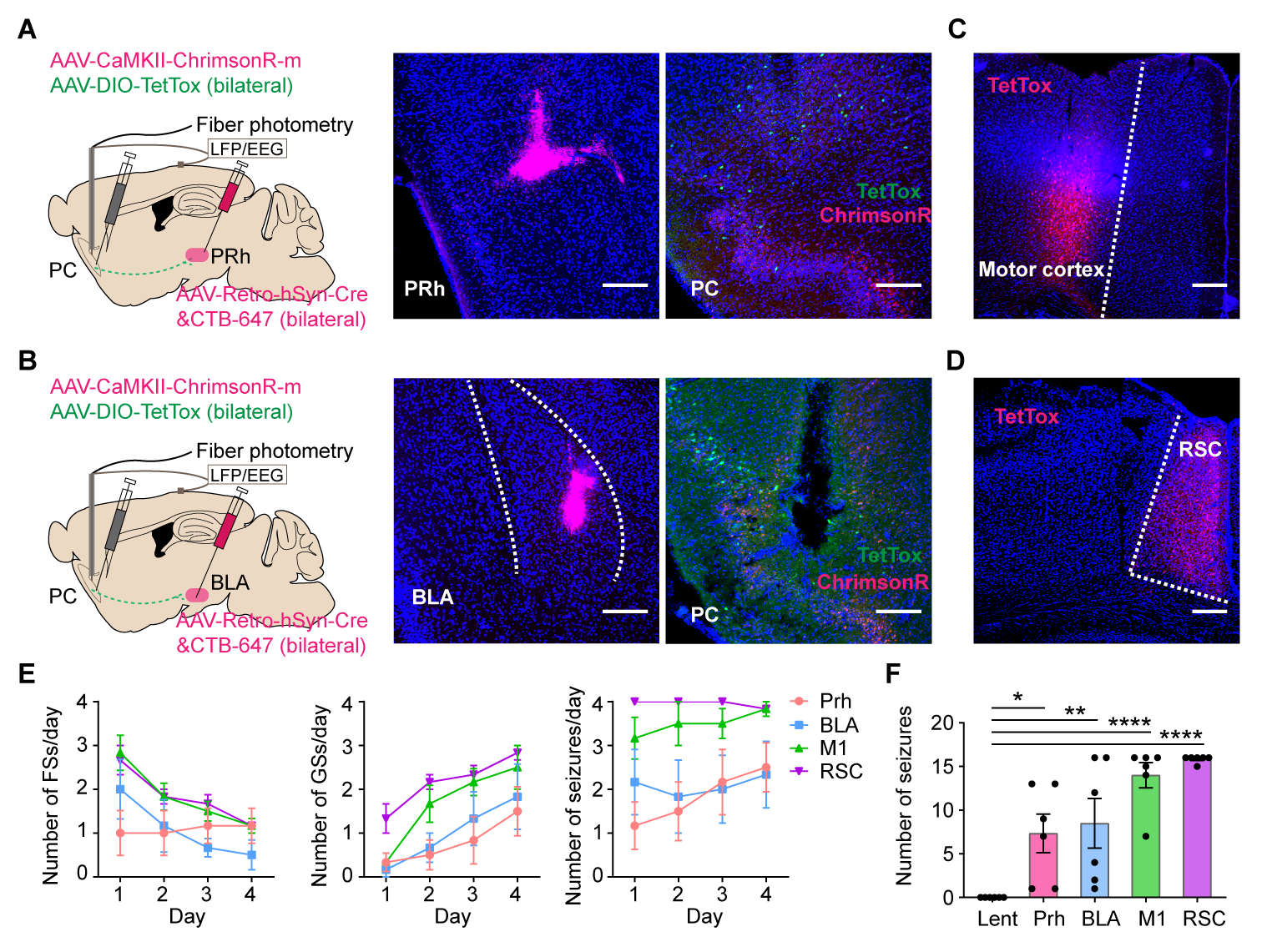

Supplement: S10 Fig — (A) Inhibition of the bilateral PC-PRh circuit using TetTox. Scale bar: 200 μm. (B) Inhibition of the bilateral PC-BLA circuit with TetTox. Scale bar: 200 μm. (C) Suppressing the bilateral motor cortex. Scale bar: 200 μm. (D) Suppressing the bilateral RSC. Scale bar: 200 μm. (E) Daily number of GSs, FSs, and total seizures; n = 6 from each group. (F) Comparison of the seizure number (one-way ANOVA followed by Dunnett’s multiple comparisons test, Lent versus Prh, P = 0.0216; Lent versus BLA, P = 0.0070; Lent versus M1, P < 0.0001; Lent versus RSC, P < 0.0001). *P < 0.05, **P < 0.01, ****P < 0.0001. The data underlying this Figure can be found in S1 Data. (TIF) [file pbio.3003577.s010.tif]

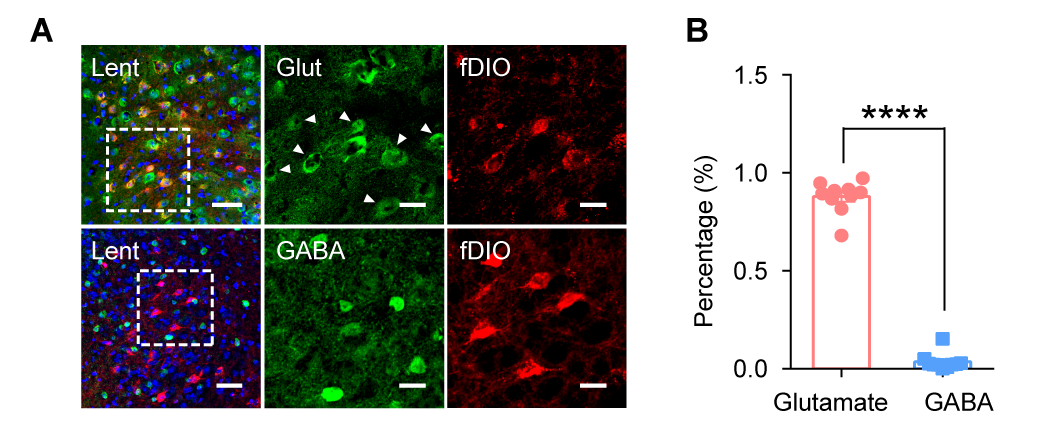

Supplement: S11 Fig — (A) Top, Glut+ and mCherry+ cells. Bottom, GABA+ and mCherry+ cells. Scale bars: 50 μm and 20 μm (enlarged images). (B) Percentage of mCherry+ cells co-labeled with Glut or GABA (Mann–Whitney test, P < 0.0001, n = 10). ****P < 0.0001. GABA: gamma-aminobutyric acid; Glut: glutamate; Lent: lateral entorhinal cortex. The data underlying this Figure can be found in S1 Data. (TIF) [file pbio.3003577.s011.tif]

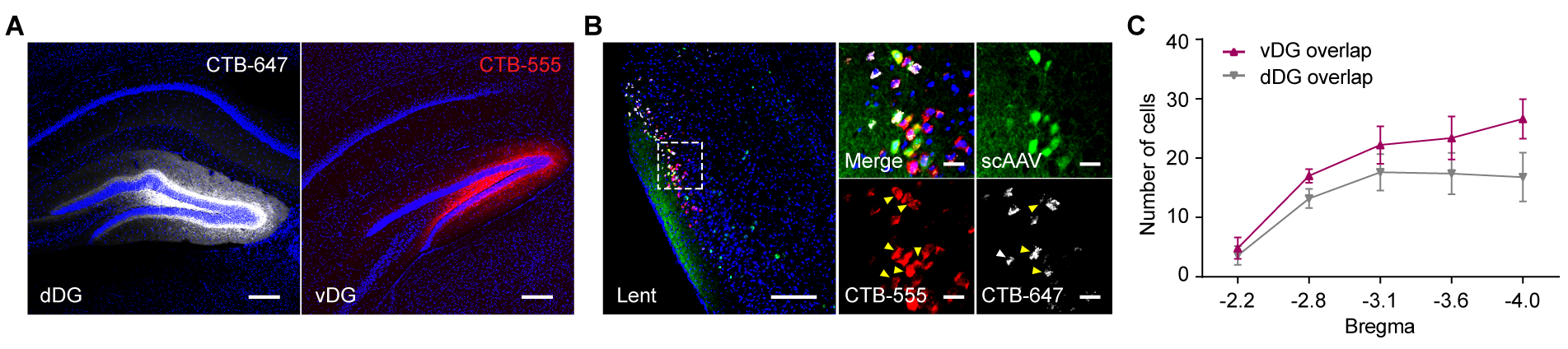

Supplement: S12 Fig — (A) Images show that CTB-647 and CTB-555 were injected into the dDG (left) and vDG (right), respectively; scale bars: 200 μm. (B) Left, the image shows GFP+ neurons co-localization with vDG-projection cells (red, CTB-555) and dDG-projection cells (white, CTB-647) in the Lent; scale bar: 100 μm. Right, an enlarged image from layers II–III of the Lent; scale bars: 10 μm. Yellow arrows indicate overlapping cells. (C) Number of GFP+ cells from the PC in layers II–III of the Lent projecting to the dDG (CTB-647+) and vDG (CTB-555+). Bregma −2.2 mm: vDG (4.800 ± 1.800); dDG (3.600 ± 1.568); Bregma −2.8 mm: vDG (17.00 ± 1.140); dDG (13.20 ± 1.625); Bregma −3.1 mm: vDG (22.20 ± 3.153); dDG (17.60 ± 3.076); Bregma −3.6 mm: vDG (23.40 ± 3.614); dDG (17.40 ± 3.473); Bregma −4.0 mm: vDG (26.60 ± 3.311); dDG (16.80 ± 4.128), n = 5. PC: piriform cortex; DG: dentate gyrus; dDG: dorsal dentate gyrus; Lent: lateral entorhinal cortex; vDG: ventral dentate gyrus. The data underlying this Figure can be found in S1 Data. (TIF) [file pbio.3003577.s012.tif]

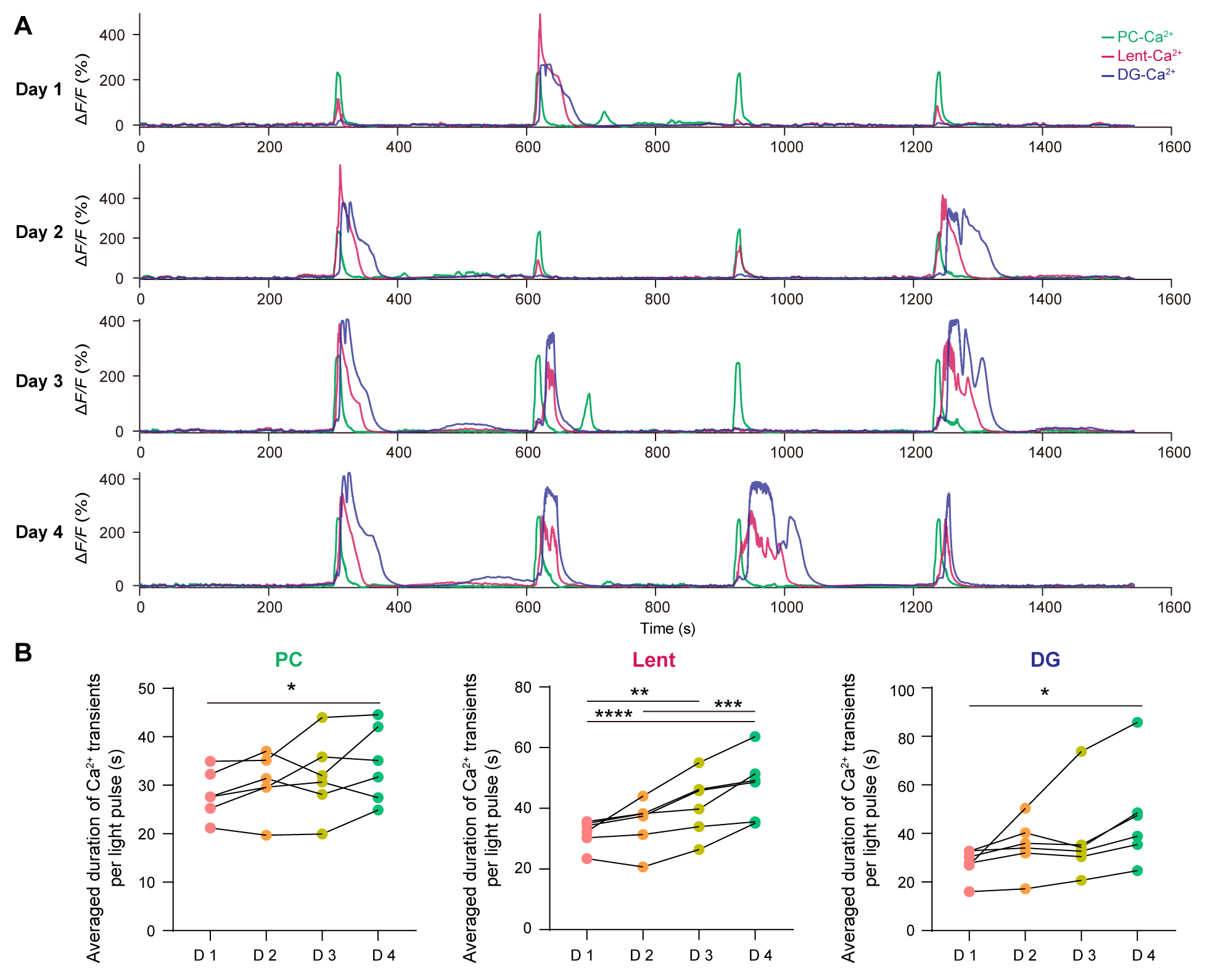

Supplement: S13 Fig — (A) Representative calcium recordings from a mouse across four consecutive days of kindling. (B) Duration of calcium response in the PC (Day 1 versus Day 4, P = 0.0130), the Lent (Day 1 versus Day 4, P < 0.0001; Day 1 versus Day 3, P = 0.0071; Day 2 versus Day 4, P = 0.0007), and the DG (Day 1 versus Day 4, P = 0.0153) over the kindling period. Repeated measures one-way ANOVA followed by Tukey’s post hoc test, n = 6. *P < 0.05, **P < 0.01, ***P < 0.001, ****P < 0.0001. The data underlying this Figure can be found in S1 Data. (TIF) [file pbio.3003577.s013.tif]
